# A systematic review and meta-analysis of the overall effects of school-based obesity prevention interventions and effect differences by intervention components

**DOI:** 10.1186/s12966-019-0848-8

**Published:** 2019-10-29

**Authors:** Zheng Liu, Han-Meng Xu, Li-Ming Wen, Yuan-Zhou Peng, Li-Zi Lin, Shuang Zhou, Wen-Hao Li, Hai-Jun Wang

**Affiliations:** 1 Department of Maternal and Child Health, School of Public Health, Peking University, Beijing, China; 2 School of Public Health, Sydney Medical School, University of Sydney, Sydney, Australia

**Keywords:** Childhood, Obesity, Prevention, Systematic review, Meta-analysis

## Abstract

**Background:**

Childhood obesity is a serious public health concern*.* School-based interventions hold great promise to combat the rising trend of childhood obesity. This systematic review aimed to assess the overall effects of school-based obesity prevention interventions, and to investigate characteristics of intervention components that are potentially effective for preventing childhood obesity.

**Methods:**

We systematically searched MEDLINE, CENTRAL and Embase databases to identify randomized- or cluster randomized- controlled trials of school-based obesity interventions published between 1990 and 2019. We conducted meta-analyses and subgroup analyses to determine the overall effects of obesity prevention programs and effect differences by various characteristics of intervention components on body mass index (BMI) or BMI Z-score of children.

**Results:**

This systematic review included a total of 50 trials (reported by 56 publications). Significant differences were found between groups on BMI (− 0.14 kg/m^2^ (95% confidence interval: − 0.21, − 0.06)) and BMI Z-score (− 0.05 (− 0.10, − 0.01)) for single-component interventions; significant differences were also found between groups on BMI (− 0.32 (− 0.54, − 0.09) kg/m^2^) and BMI Z-score (− 0.07 (− 0.14, − 0.001)) for multi-component interventions. Subgroup analyses consistently demonstrated that effects of single-component (physical activity) interventions including curricular sessions (− 0.30 (− 0.51, − 0.10) kg/m^2^ in BMI) were stronger than those without curricular sessions (− 0.04 (− 0.17, 0.09) kg/m^2^ in BMI); effects of single-component (physical activity) interventions were also strengthened if physical activity sessions emphasized participants’ enjoyment (− 0.19 (− 0.33, − 0.05) kg/m^2^ in BMI for those emphasizing participants’ enjoyment; − 0.004 (− 0.10, 0.09) kg/m^2^ in BMI for those not emphasizing participants’ enjoyment). The current body of evidence did not find specific characteristics of intervention components that were consistently associated with improved efficacy for multi-component interventions (*P* > 0.05).

**Conclusions:**

School-based interventions are generally effective in reducing excessive weight gain of children. Our findings contribute to increased understandings of potentially effective intervention characteristics for single-component (physical activity) interventions. The impact of combined components on effectiveness of multi-component interventions should be the topic of further research. More high-quality studies are also needed to confirm findings of this review.

## Introduction

Childhood overweight and obesity are global public health issues. The prevalence has increased from 16.9 to 23.8% in boys and from 16.2 to 22.6% in girls from 1980 to 2013 in developed countries, while in developing countries, the prevalence has also increased from 8.1 to 12.9% in boys and 8.4 to 13.4% in girls [1]. Childhood obesity is associated with a variety of adverse consequences [2, 3], which often persist into adulthood [4]. Therefore, prevention of childhood obesity has become one of the important public health priorities.

The main cause of childhood overweight and obesity is an energy imbalance between calories consumed and calories expended. Children spend half of their waking hours and consume at least one-third of their daily calories at school, and thus schools are being recognized as ideal vehicles for delivering obesity interventions to most children [5].

Based on the Environmental Research framework for weight Gain prevention as well as an energy balance approach [6], the goal of obesity prevention might be achieved by improvement of energy balance-related behaviors (physical activity (PA)), dietary improvement (DI)), which can be influenced by environmental influence (school policy (SP)) directly or indirectly. The direct influence reflects the “automatic, unconscious” influence of the SP on behavior. The indirect mechanism reflects the mediating role of knowledge, cognitions related to behavior (health education (HE)) in the influence of the environment on behavior. As such, a range of intervention components (PA, DI, SP, HE) have been widely used in childhood obesity prevention interventions.

Notably, a great deal of variability existed in the frequency, duration and content of intervention components [5, 7, 8]. For instance, some school-based interventions focused on increasing students’ daily physical activity [9, 10], while others only increased the frequency of physical activity by 2–3 times/week [11, 12]. Topics of health education interventions also varied. Some focused primarily on nutrition education with few physical activity or sedentary behaviors education [13, 14], some mainly on physical activity or sedentary behaviors education with few nutrition education [15, 16], while others covered both physical activity and nutrition education [9, 17]. The variety of characteristics of intervention components raises the question of what is specifically associated with intervention efficacy.

Previous reviews attempted to address question of this kind and revealed some general findings. That is, interventions covering multiple components and involving families tended to be effective [5, 6]. Three issues remained yet. First, some reviews only summarized intervention components that were commonly used in previous trials [5, 18], but they did not compare various components used in effective or non-effective trials. In other words, the identified components could be used in both effective and non-effective trials, so that the exact components uniquely related to intervention effectiveness were still unknown. Second, a previous review, focusing on the specific role of behavior change techniques, summarized “effectiveness ratio” which was determined by the ratio of intervention components used in effective trials relative to those used in both effective and non-effective trials [19]. However, the trials included in the review were weighted equally by this approach regardless of the sample size and standard error of the outcomes. Third, another review compared sub-group differences in effect sizes between trials with and without the intervention characteristics by using meta-analytic technique [20]. However, to our knowledge, this approach has not been used in specifying the effective intervention components in school-based obesity prevention interventions.

To fill the research gaps in this field, we conducted a systematic review and meta-analysis of the best available evidence from randomized controlled trials (RCT). This review aimed to firstly summarize the overall effect size of school-based obesity prevention interventions, and secondly to explore characteristics of intervention components that were associated with the improved intervention efficacy.

## Methods

### Literature search

We systematically searched three databases including MEDLINE, CENTRAL and Embase to identify RCTs of school-based obesity interventions. We included publications between January 1990 and July 2019. Our searching strategy primarily contained terms in relation to participants, interventions, body weight and study design. The full search strategy was attached in the online supporting document. The reference lists of all retrieved full text reviews were further searched for additional relevant publications. The date for our final search was July 8th, 2019.

Inclusion criteria for this review were: (1) individual- or cluster-RCT, (2) interventions implemented among students of elementary or secondary schools (aged 5~18 years), (3) studies assessing students’ body mass index (BMI) or BMI Z-score, (4) anthropometric data being collected by physical examination, (5) interventions lasting for at least 3 months, (6) intervention groups aiming for promoting healthy weight or prevention of overweight or obesity rather than treatment of overweight or obesity, (7) comparison groups being active controls, usual practice controls (maintaining “normal” school activities) or wait-list controls, (8) the English version of full-text publications available (for pragmatic reasons), as well as (9) studies providing data for meta-analyses (means, standard deviations (SDs) or 95% confidence intervals (CIs)).

Exclusion criteria included (1) studies only using questionnaires to collect the adiposity outcomes, and (2) studies specifically designed for the treatment of obesity-related diseases (e.g., type 2 diabetes or hypertension).

### Screening and data extraction

First, two reviewers (HMX; YZP) independently screened the titles and abstracts of publications obtained by the searches. Second, full texts were further identified for their eligibility. Reference lists of reviews were additionally checked for their eligibility. Discrepancies between the two reviewers (HMX; YZP) were discussed by themselves or with a third reviewer (ZL) and resolved with consensus.

The first reviewer (ZL) developed a detailed coding scheme, and the extraction items included authors, year of publication, study design, sample size, age of participants, percentages of female participants, components and characteristics of interventions, outcome measures and assessment of risk of bias. The components and characteristics of interventions were extracted from both the main papers and the intervention protocols [20–30]. Authors were further contacted for details that were not reported in the publications (in three cases). A second reviewer (HMX) independently extracted data from all the included studies, and 20% of the extracted data were double checked by the first reviewer (ZL). Disagreements in relation to data extraction were resolved by a brief discussion (kappa statistics: 0.62; in five cases).

### Assessment of risk of bias

Risk of bias of individual studies was assessed following the Cochrane guidance [31]. The assessment contains the following domains including (1) random sequence generation (*whether or not the study used a randomized sequence of assignments*), (2) concealment of the allocation sequence (*whether or not the allocation sequences were protected by adequate concealment*), (3) blinding of participants and personnel (*whether or not participants or healthcare providers were aware of intervention assignments*), (4) blinding of outcome assessment (*whether or not people who determined outcome measurements were aware of intervention assignments*), (5) incomplete outcome data (*the possibility of bias due to missing outcome data*), (6) selective outcome reporting (*whether or not the results reported were consistent with the original variables in the protocol*) and (7) other bias (*the possibility of bias not reported in the previous domains*). The leading author (ZL) was responsible for training the other author (HMX) to ensure a consistent understanding of the evaluation criteria of risk of bias between the two authors (ZL; HMX). Each domain was rated as having a high, low or unclear risk of bias. We also paid particular attention to the use of statistical methods specific to cluster-randomized trials (whether or not considering the cluster effect), and rated them in the domain of other bias.

### Data synthesis

We calculated differences in means of BMI and BMI Z-score between intervention and control groups that were reported change from baseline or follow-up BMI indices controlled for baseline measures. If the trials reported data at both immediately post-intervention and subsequent follow-ups, only the data at immediately post-intervention was included in the meta-analyses (as most of the included studies did not report the sustained effect of interventions).

As we expected considerable heterogeneity across studies, the random-effects model was used to pool the weighted results by inverse variance methods. We used the I^2^ statistic to provide a measure of heterogeneity. Results with *P* < 0.05 are reported as significant. The level of heterogeneity across studies were rated as low (I^2^ = 25%), moderate (I^2^ = 50%) or high (I^2^ = 75%). We used Stata/SE 15.0 (StataCorp) for all analyses.

### Subgroup analyses

To identify the characteristics of interventions potentially contributing to the improved effects, we first categorized interventions into those having the specified intervention components (i.e., SP, HE, PA and DI) and those without these. Then, we classified interventions into those using single or multiple components, as their effect sizes were detected as significantly different in previous reviews [6, 32]. Further, we used subgroup analyses to examine differences in effect sizes by inclusion of SP related to obesity prevention (for multi-component interventions; yes vs. no), whether or not topics in HE covering both energy input and expenditure (for both single- and multi-component interventions; yes vs. no), duration and frequency of PA (for both single- and multi-component interventions; ≥3 times/week and ≥ 10 min/time vs. < 3 times/week or < 10 min/time), whether or not including curricular PA (for both single- and multi-component interventions; yes vs. no), whether or not focusing on students’ enjoyment of PA (for both single- and multi-component interventions; yes vs. no), and whether or not including the DI component (for multi-component interventions; yes vs. no).

### Sensitivity analyses

We conducted sensitivity analyses for the following considerations:

1. If heterogeneity in the meta-analyses was moderate or high, we additionally obtained the pooled results by excluding individual studies for which the 95% CI of the intervention effect does not overlap with others.

2. We compared the pooled results obtained by all studies with those excluding individual studies at high risk of bias.

3. We grouped all comparisons according to characteristics of the study population (**sex**: exclusively boys, exclusively girls; **weight status at baseline**: not overweight or obesity, overweight or obese; **country**: middle-income countries, high-income countries). If a minimum of 2 studies (data available) was included in each group, we would further conduct sub-group analyses to investigate whether intervention effectiveness differed within sub-groups.

### Assessment of publication bias

We assessed the possibility of publication bias by drawing funnel plots. We recognized that asymmetry of funnel plots can be due to publication bias or a genuine relationship between effect size and trial size. There were a minimum of 10 studies required for the meaningful interpretation of funnel plots. We also conducted Egger’s regression test to more definitely ascertain whether publication bias was present.

## Results

### Literature screening

**Fig. 1:**
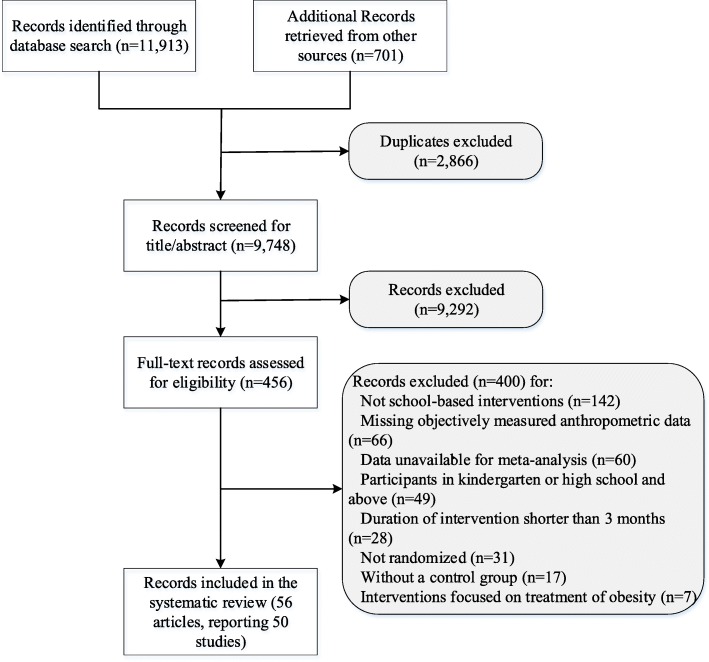
Study flow of the review

We identified 12,614 relevant records, and 2866 were excluded due to duplicates. The titles or abstracts of 9748 records were then screened and 456 full-text articles were further checked for their eligibility. Finally, 50 trials (involving 63,734 children) reported by 56 articles [9, 12–17, 33–81] that met the eligibility criteria were included in this review. The flowchart of screening process is presented in Fig. 1. The list of excluded studies is shown in Additional file 1: Table S8.

### Characteristics of included trials

Characteristics of the included trials are demonstrated in Table 1 and Additional file 1: Tables S1-S3. Most of them (*n* = 47, 94%) were cluster RCTs using the school or class as the unit of randomization. All studies had one arm as the intervention group with exception of three studies [35, 57, 64]. All studies used usual practice controls except one using an active control, in order to mitigate the potential of the Hawthorne effect [13]. A large proportion of the studies were implemented in high-income countries (*n* = 40, 80%). Most of them (*n* = 43, 86.0%) were implemented exclusively in elementary schools (mean age: 8.1 years). The follow-up period of trials ranged from 3 months to 6 years, and more than half (*n* = 32, 64%) of them maintained shorter than 12 months.

**Table 1:** Characteristics of included interventions (*n* = 50)

Category	*N*	%
Study design		
Cluster RCT	47	94.0
RCT	3	6.0
Duration of interventions, months		
3–12	32	64.0
> 12	18	36.0
Types of schools		
Elementary	43	86.0
Secondary	6	12.0
Mixed	1	2.0
Income level of country		
High-income	40	80
Middle income	10	20
Types of interventions		
Single-component (*n* = 15)		
PA	7	3.5
HE	7	3.5
SP	1	2.0
Multi-component (*n* = 35)		
PA + SP	2	3.9
HE+SP	5	9.8
PA + HE+/−SP	10	19.6
PA + DI+/−SP	3	5.9
HE+DI+/−SP	7	13.7
PA + HE+DI+/−SP	8	15.7

*RCT* randomized controlled trial, *PA* physical activity, *HE* health education, *DI* dietary improvement, *SP* school policy; “+/−”: with or without

Thirty-five (70%) interventions were multi-component while others adopted single component. HE (*n* = 7) or PA (*n* = 7) was mostly used among single-component interventions. The combinations of components mostly used in multi-component interventions were PA + HE+/−SP (*n* = 10; “+/−”: with or without), and PA + HE+DI+/−SP (*n* = 8), followed by HE+DI+/−SP (*n* = 7), HE+SP (*n* = 5), PA + DI+/−SP (*n* = 3), and PA + SP (*n* = 2).

### Assessment of risk of bias

**Fig. 2:**
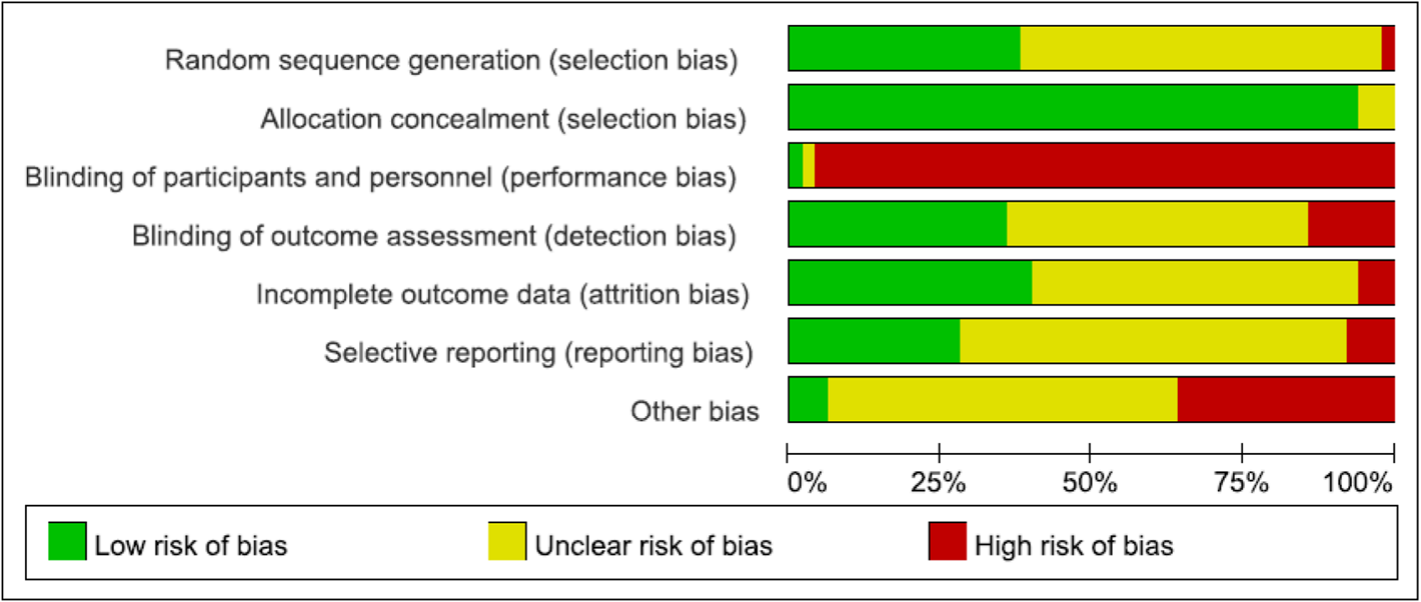
Risk of bias graph

Assessment of risk of bias was summarized in Fig. 2. Most of the trials (*n* = 47, 98%) were assessed as having a low risk of bias in allocation concealment. And most of the trials (*n* = 49, 98%) were judged as having a high risk of bias in blinding of participants and (or) personnel because it was usually not possible for interventions of this nature. Approximately half of the studies were assessed as having an unclear risk of bias due to insufficient descriptions in terms of random sequence generation (*n* = 30, 60%), blinding of outcome assessment (*n* = 25, 50.0%), incomplete outcome data (*n* = 27, 54%) or the possibility of selective reporting (*n* = 32, 64%).

### Overall effect size

**Fig. 3:**
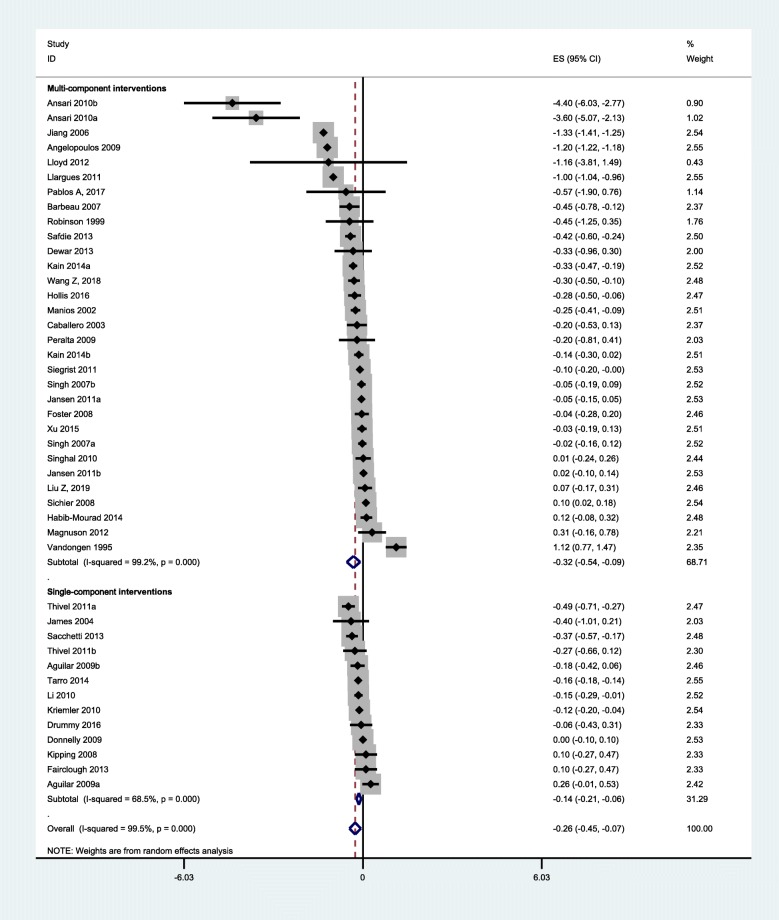
Pooled intervention effect (BMI)

**Fig. 4:**
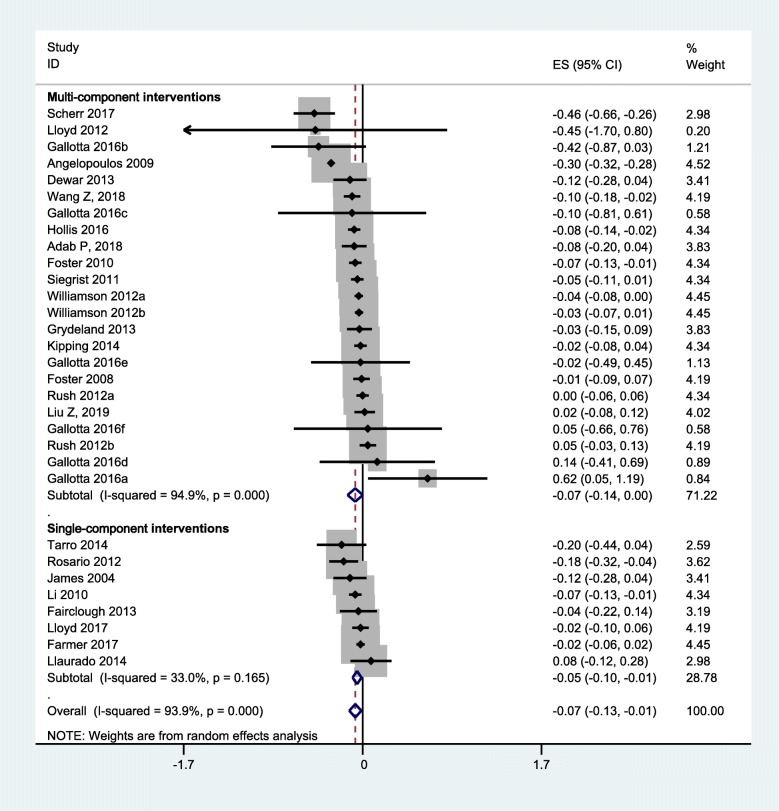
Pooled intervention effect (BMI Z-score)

Overall effect size was summarized in Figs. 3 and 4. The quantitative synthesis of the single-component interventions showed a significant, but small reduction of 0.14 (95% CI: 0.06, 0.21) kg/m^2^ in BMI, and a small reduction of BMI Z-score (0.05, 95% CI: 0.01, 0.10) compared with the control group. For the multi-component interventions, the pooled results showed a significant, but mild reduction of 0.32 (0.09, 0.54) kg/m^2^ in BMI, and 0.07 (0.001, 0.14) in BMI Z-score compared with the control group. Although the pooled effect sizes in BMI indices of multi-component interventions were slightly larger than that of single-component interventions, the differences were not statistically significant (*P* = 0.41 for BMI, *P* = 0.71 for BMI Z-score).

Findings of overall effect size were robust to the exclusion of heterogeneous studies or studies of high risk of bias (Additional file 1: Figures S1-S3). Results were also not significantly different across sex, weight status and country of the study population (Additional file 1: Table S4).

### Subgroup analyses

Subgroup analyses showed that means of BMI differed significantly by whether or not studies including curricular PA sessions or emphasizing enjoyment in PA among single-component interventions (Table 2). The pooled BMI from single-component interventions including curricular PA (*n* = 3) was − 0.30 (95% CI: − 0.51, − 0.10) kg/m^2^, while the effect size from single-component interventions not including curricular PA (*n* = 4) was − 0.04 (95% CI: − 0.17, 0.09) kg/m^2^. The pooled BMI from interventions focusing on enjoyment of participants during PA (*n* = 5) was − 0.19 (95% CI: − 0.33, − 0.05) kg/m^2^, while the effect size for interventions not emphasizing enjoyment of participants (*n* = 2) was − 0.004 (95% CI: − 0.10, 0.09) kg/m^2^. The effect sizes did not differ significantly on other intervention characteristics among single-component interventions (*P* > 0.05). Findings of subgroup analyses for single-component interventions were consistent with results from sensitivity analyses (Additional file 1: Table S6).

**Table 2:** Subgroup analyses by characteristics of single-component interventions

Outcomes	BMI			BMI Z-score		
	*N*	Mean difference, 95% CI	*P* for subgroup analyses	*N*	Mean difference, 95% CI	*P* for subgroup analyses
Characteristics of the PA component						
1) PA’s frequency and duration						
≥ 3/week and ≥ 10 min/time	5	−0.10 (− 0.22, 0.01)	0.16	–	–	–
< 3/week or < 10 min/time	2	−0.31 (− 0.57, − 0.05)		–	–	
2) Curricular PA						
Yes	3	−0.30 (− 0.51, − 0.10)	0.02	–	–	–
No	4	−0.04 (− 0.17, 0.09)		–	–	
3) PA emphasizing enjoyment						
Yes	5	−0.19 (− 0.33, − 0.05)	0.03	–	–	–
No	2	−0.004 (− 0.10, 0.09)		–	–	
Topics of HE covering both energy intake and output						
Yes	3	−0.06 (− 0.26, 0.13)	–	5	− 0.07 (− 0.16, − 0.03)	–
No	1	−0.40 (− 1.01, 0.21)		1	− 0.12 (− 0.28, 0.04)	

*CI* confidence interval, *SP* school policy, *PA* physical activity, *HE* health education, *DI* dietary improvement. “-” due to insufficient observations

Concerning multi-component interventions, subgroup analyses demonstrated that the mean BMI or BMI Z-score differed significantly by interventions emphasizing enjoyment in PA (Table 3); however, this difference was disappeared when excluding one heterogeneous study (Additional file 1: Table S5). No significant differences in effect sizes (*P* > 0.05) were detected between multi-component interventions with and without other intervention characteristics, which was consistent with results from sensitivity analyses (Additional file 1: Table S5, S7).

**Table 3:** Subgroup analyses by characteristics of multi-component interventions

Outcomes	BMI			BMI Z-score		
	*N*	Mean difference, 95% CI	*P* for subgroup analyses	*N*	Mean difference, 95% CI	*P* for subgroup analyses
Characteristics of the PA component						
1) PA’s frequency and duration						
≥ 3/week and ≥ 10 min/time	10	−0.48 (− 0.94, − 0.01)	0.63	–	–	–
< 3/week or < 10 min/time	8	− 0.29 (− 0.76, 0.19)		–	–	
2) Curricular PA						
Yes	14	−0.28 (− 0.60, 0.05)	0.19	7	−0.07 (− 0.19, 0.04)	0.26
No	5	−0.89 (−1.75, − 0.03)		2	− 0.06 (− 0.16, 0.03)	
3) PA emphasizing enjoyment						
Yes	8	−0.88 (−1.42, − 0.34)	0.02^a^	–	–	–
No	11	−0.12 (− 0.42, 0.19)		–	–	–
Topics of HE covering both energy intake and output						
Yes	15	−0.28 (− 0.57, 0.01)	0.07	8	−0.17 (− 0.29, − 0.04)	0.18
No	8	0.04 (− 0.14, 0.22)		5	−0.06 (− 0.15, 0.03)	
Inclusion of the DI component						
Yes	12	−0.22 (− 0.63, 0.18)	0.45	10	−0.07 (− 0.18, 0.04)	0.76
No	15	−0.42 (− 0.75, − 0.09)		6	−0.05 (− 0.07, − 0.03)	
Inclusion of the SP component						
Yes	18	−0.36 (− 0.63, − 0.09)	0.07	11	−0.04 (− 0.08, − 0.01)	0.52
No	7	− 0.01 (− 0.26, 0.24)		2	−0.08 (− 0.20, 0.03)	

*Abbreviations*: *CI* confidence interval, *SP* school policy, *PA* physical activity, *HE* health education

^a^Significance of the finding was disappeared when excluding the heterogeneous study (see Additional file 1: Table S5)

### Assessment of publication bias

**Fig. 5:**
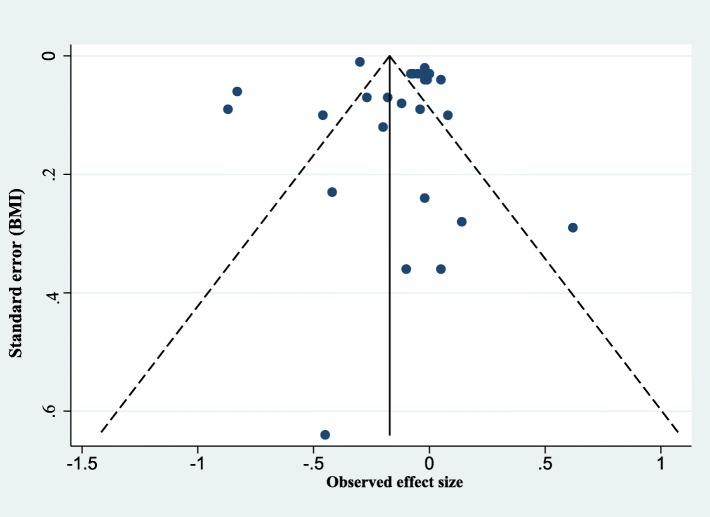
Assessment of publication bias: funnel plot

As shown in Fig. 5, the funnel plot of the observed effect showed a slightly asymmetric scatter consistent with publication bias, but *P* value for Egger’s regression test was larger than 0.05.

## Discussion

This review is one of the first to use meta-analyses and subgroup analyses to systematically review a number of more recent studies, and analyze the potentially effective characteristics of school-based interventions for preventing obesity.

### Interpretation of the study findings

This review found that emphasizing enjoyment in PA sessions was critical for single-component (PA) interventions. This finding was echoed by previous reviews suggesting that lack of motivation and pleasure of physical activity was a barrier to physical activity for children [82, 83]. Including curricular PA sessions was also found to be associated with improved efficacy of single-component (PA) interventions. This is, at least partly, explained by the fact that curricular PA sessions were usually led by physical education teachers, and thus intensity of exercise was superior to those including only extracurricular activities, after-school sessions or short activity breaks. Further, the curricular PA sessions were usually structured and compulsory for all children in a class and thus adherence could be relatively guaranteed. Significant associations between intervention components and efficacy were not consistently found in multi-component interventions. It is likely that multi-component interventions demonstrated to be effective were influenced by a combination of intervention components. The impact of combining components on intervention effectiveness should be the topic of further research.

For the current body of evidence, we did not find significant associations between dietary improvement components with improved intervention efficacy. This finding was consistent with another recent systematic review of school-based childhood obesity interventions [84]. The non-significant finding in relation to diet might be interpreted by poor adherence to diet intervention or the complex interplay of intervention components. We acknowledge that interaction analyses of intervention components (“intervention × component”) within individual studies would have provided a powerful method of understanding the complex interplay of intervention components. However, of the studies screened for this review, none reported such “intervention × component” analyses. Therefore, future obesity prevention interventions should address the specific interplay of intervention components, providing the possibility for further systematic reviews.

Findings of the study should also be interpreted in the context. The reporting of intervention characteristics (dose, frequency, and content) varied so much between trials that we were obliged to dichotomise it simply as “including the specific characteristics of component: yes/no” for the purpose of analysis, being nevertheless aware that resolution of the measure might be compromised in the process.

### Comparison with other studies

Some previous reviews of obesity interventions have attempted to address the question of “what” (characteristics or components of interventions) really works for the targeted population [5, 17, 18], but only general findings were revealed. Further, research gaps remained in relation to the weakness of methods that were used (i.e., no comparisons between effective and non-effective trials; equal weighting of the included trials). The present review not only provided an update on a recent review [5] by including several new studies, but also identified the characteristics of effective interventions through meta-analyses and subgroup analyses. Thus, this review provides important and helpful evidence of the potentially effective intervention components with different characteristics.

### Limitations and strengths of the study

Our results should be weighted cautiously considering the following limitations. First, the studies included in this review were restricted to English full-text publications found in three electronic databases. Second, the considerable level of heterogeneity was detected across studies in this review, which is relatively common among complex obesity interventions. Heterogeneity might be originated from the fidelity of the intervention and the population targeted among other factors. We have conducted sensitivity analyses to address this concern. Third, precisely evaluating the contents of some interventions is difficult and problematic due to inconsistent reporting. Future trials should be required to report interventions in accordance with TIDieR (template for intervention description and replication) [85] or other tools. Fourth, solely using BMI indices as outcome measures in this review is relatively narrow and insensitive, especially when studying PA interventions, as PA interventions might have an impact on BMI by affecting intermediate outcomes (increasing PA). We are planning to consider using behavioral outcomes in a future systematic review. Fifth, we only included RCTs in this review, which cannot address complex interplay of behaviors and real-world settings. However, RCTs are the best available approach to answer “can it work?”, as non-randomized trials might result in incomparable baseline data between the two groups, and uncontrolled trials can hardly eliminate the risk of self-selection bias. Sixth, due to the limited number of included studies as well as the limited sub-group data available for meta-analyses, we cannot investigate whether our findings of potentially effective intervention components were influenced by sex, weight status or socio-economic status of the study population. This is thus should be a potential focus for future trials, which provides a basis for the coming meta-analyses.

Despite these limitations, our study, based on a systematical review of the best available evidence from RCTs, took a first step towards distinguishing characteristics of effective school-based obesity prevention interventions. The findings of this review enable a better understanding of the effectiveness of complex school-based obesity prevention interventions. Specifically, the findings of this review suggest that school-based interventions could have significant effects on reducing students’ BMI. The effects of single-component (PA) interventions can be improved when emphasizing students’ enjoyment in physical activity, or including curricular PA sessions.

## Conclusions

Overall, school-based interventions are effective in reducing excessive weight gain of children. Findings of this review increase our understandings of potentially effective characteristics of interventions. Future high-quality studies should focus more on the interplay of intervention components, which could deepen our understandings of the complexity of obesity prevention interventions delivered in school settings.

## Supplementary information

**Additional file 1: Table S1.** Description of the included trials. **Table S2.** Description of the characteristics of the PA component for the included studies. **Table S3.** Description of the characteristics of the DI component for the included studies. **Table S4.** Differences of overall effect size by sex, weight status and country of the study population. **Table S5.** Subgroup analyses by characteristics of multi-component interventions (excluding heterogeneous studies). **Table S6.** Subgroup analyses by characteristics of single-component interventions (excluding trials assessed as high risk of bias). **Table S7.** Subgroup analyses by characteristics of multi-component interventions (excluding trials assessed as high risk of bias). **Table S8.** The list of excluded studies. **Figure S1.** Pooled intervention effect after excluding heterogeneous studies (BMI). **Figure S2.** Pooled intervention effect after excluding studies at high risk of bias (BMI). **Figure S3.** Pooled intervention effect after excluding studies at high risk of bias (BMI Z-score).

## Data Availability

The datasets analyzed during the current study are available from the corresponding author on reasonable request.

## Abbreviations

BMI: Body mass index
CI: Confidence interval
DI: Dietary improvement
HE: Health education
PA: Physical activity
RCT: Randomized controlled trial
SD: Standard deviation
SP: School policy
TIDieR: Template for intervention description and replication
