# Supplementary material for: A systematic review and meta-analysis of the overall effects of school-based obesity prevention interventions and effect differences by intervention components
Source: Int J Behav Nutr Phys Act. 2019 Oct 29;16:95. doi: 10.1186/s12966-019-0848-8 (PMC6819386; doi:10.1186/s12966-019-0848-8)
Supplement: Supplementary file 1 — Additional file 1: Table S1. Description of the included trials. Table S2. Description of the characteristics of the PA component for the included studies. Table S3. Description of the characteristics of the DI component for the included studies. Table S4. Differences of overall effect size by sex, weight status and country of the study population. Table S5. Subgroup analyses by characteristics of multi-component interventions (excluding heterogeneous studies). Table S6. Subgroup analyses by characteristics of single-component interventions (excluding trials assessed as high risk of bias). Table S7. Subgroup analyses by characteristics of multi-component interventions (excluding trials assessed as high risk of bias). Table S8. The list of excluded studies. Figure S1. Pooled intervention effect after excluding heterogeneous studies (BMI). Figure S2. Pooled intervention effect after excluding studies at high risk of bias (BMI). Figure S3. Pooled intervention effect after excluding studies at high risk of bias (BMI Z-score). [file 12966_2019_848_MOESM1_ESM.docx]

**A systematic review and meta-analysis of the overall effects of school-based obesity prevention interventions and effect differences by intervention components**

Zheng Liu^1 *^, Han-Meng Xu^1 *^, Li-Ming Wen^2^, Yuan-Zhou Peng^1^, Li-Zi Lin^1^, Shuang Zhou^1^, Wen-Hao Li^1^, Hai-Jun Wang^1^

1 Department of Maternal and Child Health, School of Public Health, Peking University, Beijing, China;

2 School of Public Health, Sydney Medical School, University of Sydney, Sydney, Australia;

* These authors contributed equally to this paper.

Corresponding author: Professor Hai-Jun Wang, Department of Maternal and Child Health, School of Public Health, Peking University, Beijing, China. Email: [whjun@pku.edu.cn](mailto:whjun@pku.edu.cn); Tel: +86-10-82805583

**Key words**: childhood; obesity; prevention; systematic review; meta-analysis

**A running title**: Intervention components for childhood obesity

**A list of the supporting document:**

1. Search strategy;

2. Table S1 Description of the included trials;

3. Table S2 Description of the characteristics of the PA component for the included studies;

4. Table S3 Description of the characteristics of the DI component for the included studies;

5. Table S4 Differences of overall effect size by sex, weight status and country of the study population;

6. Table S5 Subgroup analyses by characteristics of multi-component interventions (excluding heterogeneous studies);

7. Table S6 Subgroup analyses by characteristics of single-component interventions (excluding trials assessed as high risk of bias);

8. Table S7 Subgroup analyses by characteristics of multi-component interventions (excluding trials assessed as high risk of bias);

9. Table S8 The list of excluded studies;

10. Figure S1 Pooled intervention effect after excluding heterogeneous studies (BMI);

11. Figure S2 Pooled intervention effect after excluding studies at high risk of bias (BMI);

12. Figure S3 Pooled intervention effect after excluding studies at high risk of bias (BMI Z-score);

13. Protocol of a systematic review and meta-analysis of school-based obesity prevention interventions.

**Search strategy**

#1. 'obesity'/exp

#2. overweight:ab,kw,ti

#3. 'weight reduction'/exp

#4. adipos* OR obes*:ab,ti,kw

#5. overweight*:ab,kw,ti

#6. #1 OR #2 OR #3 OR #4 OR #5

#7. 'behavior therapy'/exp

#8. 'counseling'/exp

#9. 'counseling'/exp

#10. 'program evaluation'/exp

#11. 'social support'/exp

#12. 'exercise'/exp

#13. 'kinesiotherapy'/exp

#14. 'physical education'/exp

#15. exercise AND movement AND techniques:ab,kw,ti

#16. 'motor activity'/exp

#17. 'diet'/exp

#18. 'diet therapy'/exp

#19. 'health education'/exp

#20. 'health behavior'/exp

#21. 'health promotion'/exp

#22. 'school health service'/exp

#23. 'lifestyle'/exp

#24. (('obesity' NEAR/4 'intervention'):ab,kw,ti) OR 'program':ab,kw,ti OR'programme':ab,kw,ti

#25. 'lifestyle':ab,kw,ti OR 'life style':ab,kw,ti

#26. exercise*:ab,kw,ti

#27. physic*:ab,kw,ti AND next:ab,kw,ti AND (activity&:ab,kw,ti OR fit*:ab,kw,ti)

#28. walk*:ab,kw,ti OR jog*:ab,kw,ti OR swim*:ab,kw,ti OR ('weight':ab,kw,ti AND next:ab,kw,ti AND lift*:ab,kw,ti) OR danc*:ab,kw,ti OR 'aerobics':ab,kw,ti

#29. ((physic* OR strength* OR resist* OR 'circuit' OR 'weight' OR aerob* OR 'cross' OR 'endurance' OR structur*) NEAR/4 train*):ab,kw,ti

#30. 'behavioral':ab,kw,ti OR 'behavioural':ab,kw,ti OR (('behavior':ab,kw,ti OR 'behaviour':ab,kw,ti) AND next:ab,kw,ti AND 'modification':ab,kw,ti) OR psychoth*:ab,kw,ti OR 'psychosocial':ab,kw,ti

#31. educat*:ab,kw,ti

#32. diet*:ab,kw,ti OR 'healthy nutrition':ab,kw,ti OR (nutrition*:ab,kw,ti AND next:ab,kw,ti AND ('knowledge':ab,kw,ti OR educat*:ab,kw,ti OR therap*:ab,kw,ti OR program*:ab,kw,ti OR intervention*:ab,kw,ti))

#33. #7 OR #8 OR #9 OR #10 OR #11 OR #12 OR #13 OR #14 OR #15 OR #16 OR #17 OR #18 OR #19 OR #20 OR #21 OR #22 OR #23 OR #24 OR #25 OR #26 OR #27 OR #28 OR #29 OR #30 OR #31 OR #32

#34. #6 AND #33

#35. 'adolescent'/exp

#36. 'child'/exp

#37. 'pediatrics'/exp

#38. 'child':ab,kw,ti OR 'childs':ab,kw,ti OR children*:ab,kw,ti OR childhood*:ab,kw,ti OR childcare*:ab,kw,ti OR schoolchild*:ab,kw,ti

#39. adolescen*:ab,kw,ti

#40. paediatric*:ab,kw,ti OR pediatric*:ab,kw,ti

#41. #35 OR #36 OR #37 OR #38 OR #39 OR #40

#42. #34 AND #41

#43. 'clinical trial':ab,kw,ti

#44. (singl* OR doubl* OR trebl* OR tripl*) AND (mask*:ab,kw,ti OR blind*:ab,kw,ti)

#45. placebo*:ab,ti,kw

#46. random*:ab,kw,ti

#47. 'randomized controlled trial (topic)'/exp

#48. 'randomization'/exp

#49. 'double blind procedure'/exp

#50. 'single blind procedure'/exp

#51. 'clinical trial (topic)'/exp

#52. 'placebo'/exp

#53. #43 OR #44 OR #45 OR #46 OR #47 OR #48 OR #49 OR #50 OR #51 OR #52

#54. #42 AND #53

| **Table S1 Description of the included trials** | | | | | | | | |
| --- | --- | --- | --- | --- | --- | --- | --- | --- |
| **Author, year** | **Study design** | **Population** | **Intervention** | **Comparison** | **Main findings (Intervention)** | **Main findings (Comparison)** | **Conclusions** | **Funding source** |
| **Single-component intervention** | | | | | | | | |
| ***PA*** |  |  |  |  |  |  |  |  |
| Drummy, 2016[1] | Cluster RCT | **Country:** Ireland; **Attrition rate**: NA; **Age:** range (9-10), mean (9.5); **Female** (%): NA; **Sample size:** (provide baseline and post-intervention measures): 7 schools, 14 classes, 107 students | **7 schools, 7 classes, 54 students**  **Theory**: NA; **Content and frequency**: 5-min classroom-based activity (teacher-led warm up and MVPA), 3 times/day; **Intervention setting:** primary school; **Length**: 3 mons; **Follow-up** **duration**: 3 mons; **Target population**: students only; **Process evaluation**: NA | **7 schools, 7 classes 53 students** normal daily routine | Anthropometric assessments -**BMI**: pre (19.4 (3.5)), post (19.3 (3.3))  Behavioral assessments -**PA**: assessed by accelerometer-determined weekday MVPA (min), pre (58.7 (16.8)), post (68.2 (25.8)) | Anthropometric assessments -**BMI**: pre (18.3 (2.4)), post (18.3 (2.6))  Behavioral assessments -**PA**: assessed as the intervention group, pre (59.7 (17.5)), post (59.2 (21.4)) | Teacher led, classroom-based breaks are beneficial in increasing PA levels, but no significant changes were observed in BMI. | NA |
| Kriemler, 2010[2] | Cluster RCT | **Country:** Switzerland; **Attrition rate**: 19 (3.8%); **Age:**  mean (9.3); **Female** (%): 51.2; **Sample size:** (randomized): 15 elementary schools, 28 classes, 502 students | **9 schools, 16 classes, 297 students**  **Theory**: socio-ecological conceptual model; **Content and frequency**: PE classes (5*45min/wk), 3-5 PA breaks/day (2-5 min each), PA homework (10 min/d)); **Intervention setting:** school & home; **Length**: 9 mons; **Follow-up** **duration**: 9 mons; **Target population**: children & parents; **Process evaluation**: NA | **6 schools, 12 classes, 205 students**  PE classes (3*45min/week) | Anthropometric assessments -**BMI**: pre (17.13 (2.53)), post (17.36 (2.67))  Behavioral assessments -**PA**: assessed by accelerometer; ① MVPA (min/d) in school-pre (38 (16)), post (45 (14)); ② all day MVPA-pre (106 (36)), post (106 (34)); ③ total PA (counts/min) in school-pre (807 (276)), post (870 (217)); ④ total PA all day-pre (770 (197)), post (726 (181)) | Anthropometric assessments -**BMI**: pre (17.04 (2.63)), post (17.44 (2.89))  Behavioral assessments -**PA**: assessed as the intervention group; ① MVPA in school-pre (37 (14)), post (32 (12)); ② all day MVPA-pre (106 (33)), post (97 (34)); ③ total PA in school-pre (828 (292)), post (738 (235)); ④ total PA all day-pre (792 (204)), post (728 (225)) | A school-based PA intervention including compulsory elements improved PA and reduced adiposity in children. | the Swiss Federal Office of Sports (grant number SWI05-013), the Swiss National Science Foundation (grant number PMPDB-114401), and the Diabetes Foundation of the Region of Basel |
| Thivel, 2011[3] | Cluster RCT | **Country**: France; **Attrition rate**: NA; **Age**: range (6-10); **Female (%)**: 50.3; **Sample size**: 19 schools, 457 students | **14 schools, 229 students**  **Theory**: NA; **Content and frequency**: 120-min supervised PA & PE 2h/wk; **Intervention setting:** school only; **Length**: 6 mons; **Follow-up** **duration**: 6 mons; **Target population**: schoolchildren; **Process evaluation**: NA | **5 schools, classes, 228 students**  usual practice (PE for 2h/week) | Anthropometric assessments -**BMI**:   1) normal weight students: pre (15.62 (1.1)), post (15.55 (1.1));  2) obese students: pre (20.56 (2.6)), post (20.38 (2.9)). | Anthropometric assessments -**BMI**:   1) normal weight students: pre (15.48 (1.11)), post (15.71 (1.1));  2) obese students: pre (20.19 (1.8)), post (20.41 (1.9)) | A 6-mon school-based PA intervention did no yield positive anthropometric outcomes. | the French National Plan for Nutrition and Health (PNNS), the Comite Regional Executif des Actions de Sante d’Auvergne (CREAS), the Caisse Régionale d’Assurance Maladie d’Auvergne (CRAMA), the Appert Institutes, the town of Clermont-Ferrand, and the governing bodies of the Clermont-Ferrand school system |
| Aguilar, 2009[4] | Cluster RCT | **Country**: Spain; **Attrition rate**: 123 (11.8%); **Age**: mean (9.4 (0.7)); **Female (%)**: 51.5; **Sample size**: 20 schools, 1044 students | **10 schools, 375 students analyzed**  **Theory**: NA; **Content and frequency**: three 90-min sessions of PA/wk; **Intervention setting:** school only; **Length**: 2 years (28 wks per year); **Follow-up** **duration**: 2 years; **Target population**: schoolchildren; **Process evaluation**: NA | **10 schools, classes, 546 students analyzed**  Standard PE (provided in both the control and intervention arms) | Anthropometric assessments -**BMI**:   1) Girls: pre (18.5 (3.3)), post (19.3 (3.3));  2) Boys: pre (18.3 (3.5)), post (19.3 (3.7)) | Anthropometric assessments -**BMI**:   1) Girls: pre (18.5 (3.7)), post (19.4 (3.8));  2) Boys: pre (18.8 (3.4)), post (19.6 (3.5)) | The effectiveness of the school-based program in preventing obesity is greater in girls. | Funded mainly by the Castilla-La Mancha Health Council (grant GC03060-00). Additional funding was obtained from the Ministry of Health and Consumption, Carlos III Health Institute, Research Network in Preventive and Health Promotion Activities (Red de Investigación en Actividades Preventivas y de Promoción de Salud; grant RD06/0018/0038). |
| Donnelly, 2009[5] | Cluster RCT | **Country**: USA; **Attrition rate**: 37 (2.42%); **Age**: mean (8.3); **Female (%)**: 51.2; **Sample size**: 24 schools, 1527 students | **14 schools, 814 students**  **Theory**: NA; **Content and frequency**: Promote 90-min/wk of MVPA (about 10 min each) incorporated into academic classes; **Intervention setting:** school only; **Length**: 3 years; **Follow-up** **duration**: 3 years; **Target population**: students; **Process evaluation**: 1) delivery of PA; 2) intensity of PA; 3) teacher participation | 10 schools, 713 students  Regular classroom instruction without physically active lessons | Anthropometric assessments -**BMI**: pre (17.9 (3.1)), post (19.9 (4.1))  Behavioral assessments -**PA**: assessed by accelerometer; minutes of MVPA during intervention (98 (42.7)), etc. | Anthropometric assessments -**BMI**: pre (18.0 (3.7)), post (20.0 (4.6))  Behavioral assessments -**PA**: assessed as the intervention group; 72 (36.5)), etc. | 1. The "Physical Activity Across the Curriculum" approach may promote daily PA; 2. 75 minutes of PA activities might attenuate increases in BMI. | grant NIH NIDDK R01 061489 from the National Institute of Diabetes and Digestive and Kidney Disease, Bethesda, MD |
| Li, 2010[6] | Cluster RCT | **Country**: mainland China; **Attrition rate**: 513 (10.9%) at year 1, 580 (12.3%) at year 2; **Age**: mean (9.3 (0.7)), range (8-11); **Female (%)**: 47.7; **Sample size**: 20 schools, 4700 students | **10 schools, 2329 students**  **Theory**: NA; **Content and frequency**: 20-min of daily MVPA (2 daily 10-min sessions) exercise in the classroom; **Intervention setting:** school only; **Length**: 1 year; **Follow-up** **duration**: 2 years; **Target population**: students; **Process evaluation**: NA | **10 schools, 2371 students** Usual practice | Anthropometric assessments -**BMI**: change from pre- to post-intervention (0.56 (1.15)) -**BMI Z**: change from pre- to post-intervention (-0.05 (0.44)) | Anthropometric assessments -**BMI**: change from pre- to post-intervention (0.72 (1.20)) -**BMI Z**: change from pre- to post-intervention (0.01 (0.59)) | 20 min of daily MVPA during the school is a feasible and effective way to combat childhood obesity. | Nutricia Research Foundation |
| Sacchetti, 2013[7] | Cluster RCT | **Country**: Italy; **Attrition rate**: 69 (13.9%); **Age**: range (8-9); **Female (%)**: 48.5; **Sample size**: 26 classes, 497 students | **212 students analyzed**  **Theory**: NA; **Content and frequency**: daily 45 min of MVPA in multi-environments (schoolyard, classroom, gym); **Intervention setting:** school only; **Length**: 2 years; **Follow-up** **duration**: 2 year; **Target population**: students; **Process evaluation**: NA | **216 students analyzed** A traditional PE program | Anthropometric assessments -**BMI**: pre (18.0 (2.9)), post (19.2 (3.3))  Behavioral assessments -**PA**: assessed by self-reported questionnaire; the percentage of active children (%) changed from 11.3 to 14.6 | Anthropometric assessments -**BMI**: pre (17.8 (2.9)), post (19.4 (3.4))  Behavioral assessments -**PA**: assessed by self-reported questionnaire; 10.2 to 11.1 | The enhanced PE program was effective in improve PA and reduce excess gain of BMI. | the Italian Ministry of University and Scientific Research‐Local projects |
| ***HE*** |  |  |  |  |  |  |  |  |
| Lloyd, 2017[8] | Cluster RCT | **Country**: UK; **Attrition rate**: 80 (6.0%); **Age**: mean (9.8 (0.3)), range (9-10); **Female (%)**: 51.3; **Sample size**: 32 schools, 1324 students | **16 schools, 676 students**  **Theory**: WHO's Health Promoting Schools framework; **Content and frequency**: 1) PA workshops; 2) education sessions delivered by teachers with short homework task; 3) drama sessions; 4) setting goals to modify behaviors; **Intervention setting:** school & home; **Length**: 12 mons; **Follow-up** **duration**: 2 years; **Target population**: children & parents; **Process evaluation**: assessed both content and quality of the intervention | **16 schools, 648 students** Standard education provision | Anthropometric assessments -**BMI Z:** pre (0.32 (1.16)), post (0.35 (1.25))   Behavioral assessments -**PA**: assessed by accelerator; daily MVPA (min): pre (53.3 (16.8)), post (58.0 (22.3)); **-Nutrition:** the adjusted means of energy-dense snacks and negative food markers were lower in the intervention group than in the control group | Anthropometric assessments -**BMI Z:** pre (0.18 (1.14)), post (0.22 (1.22))  Behavioral assessments -**PA**: assessed by accelerator; daily MVPA: pre 53.9 (16.2)), post (57.0 (19.4)) **-Nutrition**: has been compared in the left column | 1. No effect of the intervention was found on preventing overweight or obesity; 2. A small effect of the intervention was found on the improvement of food intake behavior. | UK National Institute for Health Research, Public Health Research Programme |
| Llaurado, 2014[9] | Cluster RCT | **Country**: Spain; **Attrition rate**: 226 (24.7%); **Age**: mean (8.04 (0.6)), range (7-8); **Female (%)**: 60.6; **Sample size**: 16 schools, 916 students | **1 cluster (5 schools), 413 students**  **Theory**: "healthy promotion agents"; **Content and frequency**: classroom-based education covered in 12 activities (1 h/activity/ session); **Intervention setting:** school only; **Length**: 22 mons; **Follow-up** **duration**: 22 mons; **Target population**: students; **Process evaluation**: measured | **1 cluster (11 schools), 503 students** Not clearly reported in the study | Anthropometric assessments -**BMI Z**: pre (0.72), post (0.81)  Behavioral assessments -**nutrition and PA**: assessed by self-report questionnaires; improved after the intervention compared with the control group | Anthropometric assessments -**BMI Z**: pre (0.68), post (0.73)  Behavioral assessments -: has been compared in the left column | The intervention is effective in improving after-school PA in boys, but there was no change in BMI. | Diputació de Tarragona 2011 & Ajuntament d’Amposta |
| Tarro, 2014[10] | Cluster RCT | **Country**: Spain; **Attrition rate**: 411 (17.5%); **Age**: mean (8.4 (0.6)); **Female (%)**: 41.1; **Sample size**: 38 schools, 2350 students | **24 schools, 1222 students**  **Theory**: "healthy promoting agents"; **Content and frequency**: 12 classroom activities covering 8 education topics (1 hr/activity), booklets, parental activities; **Intervention setting:** school only; **Length**: 28 mons; **Follow-up** **duration**: 28 mons; **Target population**: students & parents; **Process evaluation**: NA | 14 schools, 717 students   not receiving any type of intervention | Anthropometric assessments -**BMI:** pre (31.0 (7.2)), post (18.8) -**BMI Z**: pre (0.05), post (-0.03)   Behavioral assessments -**nutrition and PA**: reported by the parent or guardian; food habits and PA changed after the intervention | Anthropometric assessments -**BMI:** pre (29.4 (7)), post (18.4) -**BMI Z**: pre (-0.11), post (0.01)  Behavioral assessments -**nutrition and PA**: has been compared in the left column | The school-based intervention delivered by "Health Promotion Agent" was effective in reducing childhood obesity prevalence in boys. | Fundació Privada Reddis, Ajuntament de Reus, Spain; Conselleria de Salut de Generalitat de Catalunya; Mercat Central de Reus, Protected Designation of Origin Siurana, Spain [DOP Siurana], La Morella Nuts, S.A. Spain; Nutrition and Health Technology Centre-TECNIO CT09-1-0019; Reus (Spain), Diputació de Tarragona, Spain |
| Kipping, 2008[11] | Cluster RCT | **Country**: England; **Attrition rate**: 207 (30.5%) for BMI measure; **Age**: mean (9.4 (0.5)), range (9-10); **Female (%)**: 38.1; **Sample size**: 19 schools, 679 students | **10 schools, 331 students**  **Theory**: social cognitive theory and behavioral choice theories of individual change; **Content and frequency**: sixteen lessons on healthy eating, PA and reducing TV reviewing taught by teachers; **Intervention setting:** school only; **Length**: 5 mons; **Follow-up** **duration**: 5 mons; **Target population**: students & teachers; **Process evaluation**: "all the teachers who were interviewed taught at least half the lessons." | **9 schools, 348 students** Being provided with the education materials after the intervention | Anthropometric assessments -**BMI:** mean difference comparing intervention to control group (0.10 (-0.27 to 0.46))  Behavioral assessments -**PA**: the interventional schools were less likely to walk/cycle to school at the end of intervention | Has been compared with the intervention group in the left column | There was no difference of BMI. | Funding was received from the Department of Health via the South West Public Health Group, South Gloucestershire Council, and DAL is funded by a Department of Health Career Scientist Award, which also funded data entry. |
| James, 2004[12] | Cluster RCT | **Country**: UK; **Attrition rate**: 210 (32.6%); **Age**: mean (8.6), range (7-11); **Female (%)**: 8.7; **Sample size**: 6 junior schools, 644 students | **15 classes, 215 students analyzed**  **Theory**: NA; **Content and frequency**: 4 sessions of focused education promoting a healthy diet and discouraging the consumption of carbonated drinks; **Intervention setting**: school only **Length**: 12 mons; **Follow-up** **duration**: 36 mons; **Target population**: students; **Process evaluation**: NA | **14 classes, 219 students analyzed** | Anthropometric assessments -**BMI:** pre (17.4 (0.6)), post (17.9 (0.7)) **-BMI Z:** pre (0.50 (0.23)), post (0.48 (0.23))  Behavioral assessments -**Nutrition:** assessed by diaries completed by the children; total carbonated drinks (the number of glasses over 3 days), pre (1.9 (0.5)), post (1.3 (0.6)) | Anthropometric assessments -**BMI:** pre (17.6 (0.7)), post (18.3 (0.8)) **-BMI Z:** pre (0.47 (0.2)), post (0.60 (0.19)  behavioral assessments -**Nutrition:** pre (1.6 (0.6)), post (1.9 (0.5)) | The intervention was effective at 12 months, but the effect was not sustained at three years. | This project was funded from unrestricted educational grants from GlaxoSmithKline, Aventis, and Pfizer and from internal resources within Bournemouth Diabetes and Endocrine Centre. |
| Fairclough, 2013[13] | Cluster RCT | **Country**: UK; **Attrition rate**: 112 (35.2%); **Age**: mean (10.6 (0.3)), range (10-11); **Female (%)**: 10.6; **Sample size**: 12 schools, 318 students | **6 schools, 166 students**  **Theory**: social cognitive theory (focusing on the interaction between social and environmental factors on behavior); **Content and frequency**: 1) teacher-led curriculum (20 weekly lessons, 60 min each), 2) learning resources, 3) homework tasks; **Intervention setting**: school & home; **Length**: 5 mons; **Follow-up** **duration**: 30 weeks; **Target population**: parents, children and teachers; **Process evaluation**: NA | **6 schools, 152 students**  Receiving normal instruction | Anthropometric assessments -**BMI:** between-group differences between baseline and post-intervention (0.10 (-0.37, 0.38)) **-BMI Z:** (-0.04 (-0.22, 0.15))  Behavioral assessments -**PA:** assessed by accelerometers; LPA, min/day (5.14 (-10.29, 20.57)), MPA (1.67 (-7.35, 10.68)), VPA (2.85 (-1.64, 7.35))  **-Nutrition:** assessed by a 24 hr recall food intake questionnaire; no sig. intervention effects were observed for previous day breakfast, fruit and vegetable intake. | Has been compared with the intervention group in the left column | 1. The intervention positively influenced body size outcomes and light PA; 2. The effect was most influential among overweight or obese children and girls. | NA |
| Rosario, 2012[14] | Cluster RCT | **Country**: Portugal; **Attrition rate**: 170 (36.6%); **Age**: 8.3 (1.2); **Female (%)**: 51.5; **Sample size**: 7 schools, 464 students | **3 schools, 233 students**  **Theory**: the Health Promotion Model and the social cognitive theory; **Content and frequency**: 12 sessions of nutrition educational program delivered by their own teachers (3 hr each); **Intervention setting**: school only; **Length**: 6 mons; **Follow-up** **duration**: 6 mons; **Target population**: teachers & children; **Process evaluation**: "The implementation of the program occurred as planned." | **4 schools, 231 students** | Anthropometric assessments **-BMI Z:** pre (0.84 (1.07)), post (0.90 (0.97))  Behavioral assessments -**PA:** assessed by questionnaire reported by parents, and then researchers divided the population into four activity classes (sedentary group, low activity group, moderately active group and vigorously active group) **-Nutrition:** assessed by one day 24-h dietary recall obtained by nutritionists and/or trained interviewers  PA and energy intake was not sig changed compared with control group. | Anthropometric assessments **-BMI Z:** pre (0.66 (1.12)), post (0.92 (1.0))  Behavioral assessments (see the left column) | The program, delivered by the in-service teachers is effective in preventing overweight and obesity of schoolchildren. | NA |
| ***SP*** |  |  |  |  |  |  |  |  |
| Farmer, 2017[15] | Cluster RCT | **Country**: New Zealand; **Attrition rate**: 210 (25%); **Age**: 8.0 (1.1); **Female (%)**: 53.7; **Sample size**: 16 schools, 840 students | **8 schools, 418 students**  **Content**: redesigned play environment (1) promoting greater challenges, 2) adding more dynamic equipment, 3) relaxing the rules); **Intervention setting**: school only; **Length**: one year; **Follow-up** **duration**: two years; **Process evaluation**: NA | **8 schools, 422 students** Not change anything in their school play spaces | Anthropometric assessments **-BMI:** pre (17.4 (2.8)), post (19.0 (3.7)); **-BMI Z:** pre (0.69 (1.11)), post (0.78 (1.16))   Behavioral assessments -**PA:** assessed by accelerometer; MVPA whole day (min): pre (67 (23)), post (62 (23)) | Anthropometric assessments **-BMI:** pre (17.4 (2.7)), post (18.7 (3.4)); **-BMI Z:** pre (0.73 (1.07)), post (0.78 (1.12))  Behavioral assessments -**PA:** pre (67 (23)), post (65 (23)) | Altering the school play environment (promoting greater risk and challenge) did not increase PA, nor alter body weight. | the Health Research Council of New Zealand and the Otago Diabetes Research Trust |
| **Multi-component intervention** | | | | | | | | |
| ***PA+SP*** |  |  |  |  |  |  |  |  |
| Hollis, 2016[16] | Cluster RCT | **Country**: Australia; **Attrition rate**: 99 (9%) at 12 mons; 165(14%) at 24 mons; **Age**: mean (12), range (12-13); **Female (%)**: 52.8; **Sample size**: 10 schools, 1150 students | **5 schools, 505 students**  **Theory:** social cognitive theory and socio-ecological theory; **Content and frequency:** multi-component PA (1) curriculum, 2) school environment, 3) parents and community)**; Intervention setting:** school only; **Length:** 24 mons; **Follow-up duration:** 24 mons; **Target population:** teachers, students, community PA providers and parents; **Process evaluation: "**All intervention implementation strategies were delivered as planned.**"** | **5 schools, 645 students** Following usual PE and sport programs | Anthropometric assessments **-BMI:** pre (19.90 (3.59)), post (21.64 (4.06)); **-BMI Z:** pre (0.54 (1.11), post (0.65 (1.12))  Behavioral assessments -**PA:** assessed by accelerometer; the percentage of MVPA≥60min/d: pre (207 (33%)), post (137 (34%)) | Anthropometric assessments **-BMI:** pre (20.19 (3.81)), post (21.90 (4.33)); **-BMI Z:** pre (0.58 (1.16)), post (0.72 (1.09))  Behavioral assessments -**PA:** pre (207 (33%)), post (90 (28%)) | The intervention achieved reductions in adiposity among adolescents from socio-economically disadvantaged communities. | the NSW Ministry of Health, Heath Promotion Demonstration grant scheme |
| Ansari, 2010[17] | Individual RCT | **Country**: Egypt; **Attrition rate**: NA; **Age**: mean (15.6); **Female (%)**: 56.3; **Sample size**: 160 students | **80 students**  **Theory:** NA; **Content and frequency:** an 'afterschool' one additional hr of exercise 3 times/wk; **Intervention setting:** school only; **Length:** 3 mons; **Follow-up duration:** 3 mons; **Target population:** students; **Process evaluation:** NA | **80 students** The 'normal' exercise schedule provided by the school | Anthropometric assessments **-BMI:  1) for boys:** pre (20.9 (4.1)), post (19.7 (3.1)); **2) for girls:** pre (21.6 (4.5)), post (20.3 (3.6)) | Anthropometric assessments **-BMI:  1) for boys:** pre (21.2 (3.6)), post (23.3 (3.5)); **2) for girls:** pre (21.4 (3.8)), post (24.7 (3.7)) | The PA program was effective in preventing childhood obesity. | NA |
| ***HE+SP*** |  |  |  |  |  |  |  |  |
| Robinson, 1999[18] | Cluster RCT | **Country**: USA; **Attrition rate**: 6 (3.0%); **Age**: mean (8.9); **Female (%)**: 46.9; **Sample size**: 2 schools, 198 students | **1 school, 95 students**  **Theory:** social cognitive theory; **Content and frequency:** 18-lesson, 6-mon class curriculum; **Intervention setting:** school & home; **Length:** 6 mons; **Follow-up duration:** 6 mons; **Target population:** teachers & students; **Process evaluation:** NA | **1 school, 103 students**  assessments-only control | Anthropometric assessments **-BMI:** pre (18.38 (3.67)), post (18.67 (3.77))  Behavioral assessments -**PA**: reported by children and parents; no sig. between-group difference in MVPA -**Nutrition:** reported by children and parents; compared with the control group, children in the intervention group reduced meals eaten in front of the TV | Anthropometric assessments **-BMI:** pre (18.10 (3.77)), post (18.81 (3.76))  Behavioral assessments -**PA**:  -**Nutrition**: (has been compared with the intervention group in the left column) | Reducing sedentary time is a promising approach to prevent childhood obesity. | This work was funded by a grant from the American Heart Association, California Affiliate, and by grant RO1 HL54102 from the National Heart, Lung, and Blood Institute, Bethesda, Md. |
| Sichieri, 2008[19] | Cluster RCT | **Country**: Brazil; **Attrition rate**: 213 (18.7%); **Age**: mean (10.9), range (9-12); **Female (%)**: 52.5; **Sample size**: 22 schools, 47 classes, 1140 students | **23 classes, 526 students**  **Theory:** NA; **Content and frequency:** a healthy lifestyle education encouraging water instead of beverage consumption (10 1-hr sessions); **Intervention setting:** school only; **Length:** 7 mons; **Follow-up duration:** 7 mons; **Target population:** students; **Process evaluation:** NA | **24 classes, 608 students** Two 1-hr general sessions on health issues and printed general advices concerning healthy diets | Anthropometric assessments **-BMI:** mean change of 'intervention-control' (0.10, 95% CI (-0.06, 0.10))  Behavioral assessments -**Nutrition:** measured through one 24-hr recall; sugar-sweetened beverages and juice intake/class (ml/d); mean change of 'intervention-control': -56.0 (-119.0, -7.0) | Has been compared with the intervention group in the left column | The intervention by decreasing sugar-sweetened beverages intake significantly reduced BMI among overweight children, and primarily among girls. | the Brazilian National Research Council – CNPq (Grant number: 500404/2003-8 – CNPq) |
| Williamson, 2012[20] | Cluster RCT | **Country**: America; **Attrition rate**: 441 (21.4%) at 18-mon 627 (30.4%) at 28-mon; **Age**: mean (10.5 (1.2)); **Female (%)**: 58.4; **Sample size**: 17 school clusters, 2060 students | **Intervention1 (environmental modification):** 5 clusters, 713 students; **Intervention2 (environmental modification & classroom and internet education):** 6 clusters, 760 students **Theory:** "adding additional education and social support for changing behaviors"; **Intervention setting:** school only; **Length:** 28 mons; **Follow-up duration:** 28 mons; **Target population:** students (for Intervention1); students & teachers & parents (for intervention2); **Process evaluation:** assessed by questionnaires and observations | **6 clusters, 587 students**  Control | Intervention1 & Intervention2 were combined.  Anthropometric assessments **-BMI Z:** the change of 'intervention-control' difference; for boys: -0.03 (-0.08); for girls:  -0.04 (-0.08);  Behavioral assessments -digital photography of **food selections and food intake:** changes in dietary fat intake were larger in the intervention group than the control group; -self-reported **PA:** not sig. | Has been compared with the intervention group in the left column | 1. The school-based program did no influence excessive weight gain; 2. Addition of a classroom/internet program did impact PA in overweight children. | the National Institute for Child Health and Human Development of the National Institutes of Health (R01 HD048483) and the U.S. Department of Agriculture (58-6435-4-90) |
| Llargues, 2011[21] | Cluster RCT | **Country**: Spain; **Attrition rate**: 89 (14.9%) for anthropometric data; **Age**: mean (6.03 (0.3)); **Female (%)**: 46; **Sample size**: 16 schools, 509 students analyzed | **8 schools, 272 students analyzed  Theory: "**the school children are actors to operate over their environment**"**; **Intervention setting:** mainly school; **Content and frequency:** 1) activities about healthy food habits and PA in classroom 3hr/wk; 2) hand out healthy recipes for the families; 3) give out materials on healthy foods and about promoting healthy PA; 4) provide equipment for games **Length:** 2 years; **Follow-up duration:** 2 years; **Target population:** students & families; **Process evaluation:** NA | **8 schools, 237 students analyzed** control | Anthropometric assessments -**BMI:** pre (16.9 (2.3)), post (17.9);  Behavioural assessments-**Food habits** were assessed by a food frequency questionnaire: the proportion of children that ate a second piece of fruit increased; -**PA** was self-reported: the proportion of children that took part in an after-school PA increased | Anthropometric assessments -**BMI:** pre (16.4 (2.7)), post (18.3);  Behavioural assessments- **Food habits:** the weekly consumption of fish was reduced | The educational intervention was effective in lessening the increase in child obesity. | This study was supported by Observatori de la Salut Carles Vallbona, Fundació Hospital Asil de Granollers, Public Health Department, Granollers City Council, Primary Health Subdivision (PCS) Granollers–Mollet, Catalan Institute of Health and by Health Department, Generalitat de Catalunya, Spain. |
| Wang Z 2018 | Cluster RCT | **Country**: Mainland China; **Attrition rate**: 233 (2.4%); **Age**: mean (10.5), SE (0.02); **Female (%)**: 46.9; **Sample size**: 48 schools, 9858 students | **24 schools, 5275 students**  **Theory:** NA; **Intervention setting:** school & home; **Content and frequency:** classroom curricula (45 min for each classroom curriculum, delivered monthly)**;** school environment support**;** family involvement; fun programs/events;  **Length:** 1 year;  **Follow-up duration:** 1 year;  **Target population:** children and parents;  **Process evaluation:** NA. | **24 schools, 4583 students**  control | Anthropometric assessments -**BMI:** pre (19.3 (0.05)), post (19.5 (0.05));  -**BMI Z**: pre (0.5 (0.02)), post (0.5 (0.02));  Behavioural assessments  **-PA:** moderate PA time assessed by validated questionnaire; pre: mean (899.6), SE (9.96); post: mean (967.7), SE (9.34) | Anthropometric assessments -**BMI:** pre (19.3 (0.05)), post (19.7 (0.06));  -**BMI Z**: pre (0.5 (0.02)), post (0.6 (0.02));  Behavioural assessments  **-PA:**  moderate PA time assessed by validated questionnaire; pre: mean (919.9), SE (13.35); post: mean (938.6), SE (13.07); | This large community-based PA intervention was effective in promoting PA and preventing obesity among the general student population in Nanjing, China. | The study (both the research project and intervention) was supported by Nanjing  Medical Science and Technique Foundation (ZDX12019), China. Zhengqi Tan, Drs  Youfa Wang and Hong Xue’s efforts were partially supported by the National Institute  of Health (NIH, U54 HD070725). |
| ***PA+HE+/-SP*** |  |  |  |  |  |  |  |  |
| Jansen, 2011[22] | Cluster RCT | **Country**: Netherland; **Attrition rate**: 206 (7.9%); **Age**: mean (9.2), range (6-12); **Female (%)**: 50.8; **Sample size**: 20 schools, 2622 students | **10 schools, 1240 students   Theory:** behavioral and ecological models; **Intervention setting:** school & home; **Content and frequency:** 1) 3 PE sessions/wk, 2) additional play and sports activity outside school hours, 3) an education program **Length:** 8 mons; **Follow-up duration:** 8 mons; **Target population:** children and parents; **Process evaluation:** NA | **10 schools, 1382 students** Continuing with usual curriculum (2 PE sessions/wk) | Anthropometric assessments -**BMI:**  1) Grade 3-5: pre (17.07 (2.76)), post (17.49 (3.00)); 2) Grade 6-8: pre (19.60 (3.98)), post (20.36 (4.24)) | Anthropometric assessments -**BMI:**  1) Grade 3-5: pre (17.07 (2.79)), post (17.57 (3.08)); 2) Grade 6-8: pre (19.06 (3.83)), post (19.77 (4.09)) | The school-based program focused on PA is effective in reducing childhood obesity. | NA |
| Manios, 2002[23] | Cluster RCT | **Country**: Greece; **Attrition rate**: NA; **Age**: range (5.5-6.5); **Female (%)**: NA; **Sample size**: 2 counties, 1046 students | **1 county, 602 students   Theory:** social learning theory; **Intervention setting:** school & home; **Content and frequency:** 1) providing multi-component workbooks for students, 2) providing teaching aids (posters, manuals etc.) for teachers, 3) 13-17 hr of classroom material about nutrition/year, 4) 4-6 hr/year of PA component delivered by PE instructors; **Length:** 72 mons; **Follow-up duration:** 72 mons; **Target population:** students & teachers; **Process evaluation:** NA | **1 county, 444 students** continuing with the usual PE classes | Anthropometric assessments -**BMI:** pre (16.3 (2.3)), post (19.9 (3.9))  Behavioral assessments- -**MVPA** (min/day) was assessed by using a standardized activity interview; pre (55.2 (116.0)), post (338.3 (361.5)); -**Food frequency** was reported by parents in a questionnaire; notable differences were observed between the intervention and the control group, i.e., the increase in the average energy intake was higher in control than intervention. | Anthropometric assessments -**BMI:** pre (16.3 (2.2)), post (20.5 (4.1))  Behavioral assessments- -**MVPA** (min/day): pre (74.6 (133.7)), post (244.2 (300.6));  -**Food frequency** | This 6-year intervention has the potential to lead to a healthier lifestyle and prevent childhood obesity. | Kellogg’s, the Greek Ministry of Sports and the Greek Ministry of Education |
| Peralta, 2009[24] | Individual RCT | **Country**: Australia; **Attrition rate**: 1 (3.0%); **Age**: mean (12.5 (0.4)), range (12-13); **Female (%)**: 0; **Sample size**: 33 students | **16 students   Theory:** Social Cognitive Theory; **Intervention setting:** school; **Content and frequency:** 1) one 60-min curriculum session/week, 2) two 20-min lunchtime PA sessions/wk; **Length:** 6 mons; **Follow-up duration:** 6 mons; **Target population:** students, teachers & parents; **Process evaluation:** included to assess fidelity, dose, reach and exposure of the intervention components | **17 students**   A general fitness program | Anthropometric assessments -**BMI:** pre (22.8 (4.1)), post (23.1 (4.2))  Behavioral assessments- -**MVPA (min/day)** assessed by accelerometer; 1) weekday: pre (88.1 (46.9)), post (112.0 (49.5)); 2) weekend: pre (39.2 (18.4)), post (53.8 (42.1)) -**Food frequency** assessed by a self-report (students) food frequency questionnaire: 1) average weekly consumption of sweetened beverages (250 ml): pre (14.3 (3.8)), post (14.8 (3.3)); 2) average weekly consumption of fruit (servings): pre (30.7 (9.3)), post (30.7 (9.4)) | Anthropometric assessments -**BMI:** pre (20.4 (4.1)), post (21.0 (4.1))  Behavioral assessments- -**MVPA**; 1) weekday: pre (73.7 (30.5)), post (92.9 (45.5)); 2) weekend:  pre (42.4 (33.5)), post (70.9 (53.6)) -**Food frequency:** 1) sweetened beverages: pre (15.0 (4.7)), post (15.6 (4.2)); 2) fruit intake: pre (30.6 (9.7)), post (27.6 (9.0)) | The multifaceted intervention was feasible, acceptable and potentially effective to prevent unhealthy weight gain among adolescent boys. | The broader intervention school community (partly funding) |
| Vandongen, 1995[25] | Cluster RCT | **Country**: Australia; **Attrition rate**: NA; **Age**: range (10-12); **Female (%)**: NA; **Sample size**: 30 schools, 1147 students | **Theory:** NA; **Intervention setting:** school & home; **Content and frequency:** 1) **5 schools, 73 students**: the school-based nutrition program: 10 1-hr lessons aiming to improve knowledge, attitude and eating habits;  2) **5 schools, 75 students**: the fitness education program: 6 30-min classroom sessions;  3) **5 schools, 72 students**: Fitness+school nutrition;  4) **5 schools, 54 students**: School nutrition+home nutrition (delivering 5 nutrition messages using comics); **Length:** 9 mons; **Follow-up duration:** 9 mons; **Target population:** teachers & students (for the school component), teachers, students & parents (for the school+home); **Process evaluation:** NA | **5 schools, 63 students** No nutritional or physical fitness programs were introduced. | Anthropometric assessments -**BMI:**  1) the school nutrition: pre (18.0 (17.5, 18.5), post (18.5 (17.9, 19.0); 2) the fitness program: pre (18.2 (17.7, 18.7)), post (18.2 (17.6, 18.8)); 3) fitness+school nutrition: pre (18.1 (17.5, 18.7)), post (18.6 (17.9, 19.3)); 4) school nutrition+home nutrition: pre (18.2 (17.4, 19.0)), post (18.4 (17.6, 19.2))  Behavioural assessments- -**Dietary intake was assessed by describing and estimating quantities of foods consumed over the following 2 weekdays by students:** 1) fat intake: in girls, decreased sig. in the school+home nutrition group; 2) fiber intake: in girls, increased in the school+home nutrition group and the fitness group;  3) sugar intake: in boys, reduced in the fitness, fitness+school nutrition, and school+home nutrition groups. | Anthropometric assessments -**BMI:** pre (18.1 (17.5, 18.6)), post (18.2 (17.6, 18.8))  Behavioral assessments- -**Dietary intake:** has been compared in the left column. | Programs should differ between boys and girls. | The National Heart Foundation of Australia (WA Division) |
| Siegrist, 2011[26] | Cluster RCT | **Country**: Germany; **Attrition rate**: 102 (12.3%); **Age**: mean (8.4 (0.7)); **Female (%)**: 48.3; **Sample size**: 8 schools, 826 students | **4 schools, 486 students  Theory:** NA; **Intervention setting:** school & home; **Content and frequency:** 1) 10 health-related lessons for students, 2) 2-3 health-related lessons for parents and teachers, 3) providing newsletters on health issues for parents and teachers; **Length:** 12 mons; **Follow-up duration:** 12 mons; **Target population:** students & teachers & parents; **Process evaluation:** NA | **4 schools, 340 students  (**2-3)*45 min/wk PE lessons given by usual classroom teachers | Anthropometric assessments -**BMI:** pre (17.4 (2.9)), post (18.1 (3.2)) -**BMI Z**: pre (0.15 (1.04)), post (0.21 (1.05))  Behavioral assessments -**PA:** the number of days in which children spent at least 60 min per day being physically active reported by questionnaire; pre (4.6 (2.0)), post (5.1 (2.0)) | Anthropometric assessments -**BMI:** pre (17.3 (3.0)), post (17.9 (3.3)) -**BMI Z**: pre (0.08 (1.07)), post (0.09 (1.10))  Behavioral assessments -**PA:** pre (4.4 (2.1)), post (4.5 (2.1)) | The PE program increased the PA of students in the intervention group, although the effect was no sig. | The Bavarian State Ministry of the Environment and Public Health (Gesund. Leben. Bayern.) (321g-G8203.1-2005/68-36) |
| Jiang, 2006[27] | Cluster RCT | **Country**: mainland China; **Attrition rate**: NA; **Age**: mean (8.3 (1.5)); **Female (%)**: 47.3; **Sample size**: 5 schools, 2489 students | **2 schools, 1029 students  Theory:** NA; **Intervention setting:** school & home; **Content and frequency:** nutrition education (once per semester for children & parents, once per fortnight for teachers, an extra meeting for children who were overweight or obese and their parents once per semester), PA (overweight and obese children running for 20min/weekday after class); **Length:** 3 years; **Follow-up duration:** 3 years; **Target population:** students, teachers & parents; **Process evaluation:** assessed the attendance rates | **3 schools, 1396 students**  Following usual health and PE curriculum | Anthropometric assessments -**BMI:** pre (17.6 (2.9)), post (18.2 (2.6)) | Anthropometric assessments -**BMI:** pre (17.5 (2.7)), post (20.3 (3.4)) | An intervention focused on nutrition education and PA could reduce the prevalence of overweight and obesity in schoolchildren in Beijing. | NA |
| Magnuson, 2012[28] [47] | Cluster RCT | **Country**: Iceland; **Attrition rate**: 67 (20.9%); **Age**: mean (7.4 (0.3)); **Female (%)**: 46.1; **Sample size**: 6 schools, 321 students | **3 schools, 151 students  Theory:** social cognitive theory (the bridge between theory: encouraging teachers to become the implementers of change needed to positively affect the children's lifestyles); **Intervention setting:** school & home; **Content and frequency:** PA (more frequent outdoor teaching, organized fieldtrips, promotion of active commute to and from school, one extra PE lesson/week (three 40-min sessions/wk)) & dietary intervention (dietary knowledge, awareness, preferences/taste, self-efficacy and parental influence; focus was on fruit and vegetable intake); **Length:** 2 years; **Follow-up duration:** 2 years; **Target population:** teachers & students; **Process evaluation:** teachers helding a log-book to keep track of children's performance | **3 schools, 170 students** General curriculum-based PA (two compulsory 40-min sessions/wk) | Anthropometric assessments -**BMI:** pre (16.0 (1.8)), post (17.4 (2.2))  Behavioral assessments -**PA:** assessed by accelerometers; "children in the intervention schools were sig. more active after 1-year of intervention compared to the control, but there was no sig. difference in either volume or intensity of PA after 2-year of intervention; "boys were more active than girls". | Anthropometric assessments -**BMI:** pre (16.7 (2.1)), post (17.5 (2.7))  -**PA:** has been compared with the intervention group in the left column | 1. The intervention did not have statistically significant effect on BMI; 2. The PA at school was increased after 1-year of intervention, but was not sustained to the end (after 2 years); 3. The effect of promoting PA was more pronounced among boys. | The study was primarily funded by the Icelandic Centre for Research (RANNIS), but also supported by the city of Reykjavik, the Ministry of Education, Science and Culture and BRIM Seafood. |
| Dewar, 2013[29] [48] | Cluster RCT | **Country**: Australia; **Attrition rate**: 67 (37.6%); **Age**: mean (13.2 (0.5)), range (12-14); **Female (%)**: 100; **Sample size**: 12 schools, 357 students | **6 schools, 178 students  Theory:** social cognitive theory; **Intervention setting:** school & home; **Content and frequency:** enhanced school sport sessions (40*90 mins), lunchtime PA sessions (30*30 mins), nutrition workshops (3*90 mins), interactive educational seminars (3*30 mins), pedometers for self-monitoring (9 mons), student handbooks (10 wks), parent newsletters (1 school term), and text messages (once weekly: 40 wks; twice weekly: 10 wks); **Length:** 12 mons; **Follow-up duration:** 24 mons; **Target population:** teachers, students & parents; **Process evaluation:** A detailed process evaluation was conducted (attendance/reach, intervention fidelity and program satisfaction) | **6 schools, 179 students** Provided with equipment packs and intervention following the completion of 24-mon assessments. | Anthropometric assessments -**BMI:** pre (22.7 (4.7)), post (23.3 (4.7)); -**BMI Z:** pre (0.82 (1.12)), post (0.76 (1.16))  Behavioral assessments -**PA:** assessed by accelerometers; MVPA (min/d): pre (33.5 (20.5 to 40.1)), post (21.5 (15.9 to 28.9)) -**Nutrition**: assessed by self-reported food frequency questionnaire; daily energy intake did no differ between the intervention group and the control group after the intervention. | Anthropometric assessments -**BMI:** pre (22.6 (4.5)), post (23.4 (4.7)); -**BMI Z:** pre (0.78 (1.16)), post (0.81 (1.17))  Behavioral assessments -**PA:** pre (32.0 (24.7 to 42.1)), post (25.0 (16.5 to 41.7)) -**Nutrition**: has been compared in the left column | Intervention effects on BMI, BMI Z and PA were not sig. | grant DP1092646 from the Australian Research Council |
| Adab P, 2018 | Cluster RCT | **Country**: UK; **Attrition rate**: 1 school (1.9%), 143 students (10.3%); **Age**: mean (6.3), SD (0.3); **Female (%)**: 48.9; **Sample size**: 54 schools, 1392 students | **26 schools, 662 students**  **Theory:** behavior change techniques**;**  **Intervention setting:** school and home;  **Content and frequency: Length:** 12 months;  **Follow-up duration:** 30 months;  **Target population:** children and their families;  **Process evaluation:** a variety of methods were used (e.g., interview)**.** | **28 schools, 735 students**  **Usual practice** | Anthropometric assessments:  -**BMI Z score:** pre-mean (0.23), SD (1.2), post-mean (0.34), SD (1.34)  Behavioural assessments:  **-PA**, assessed by energy expenditure (kj/kg/day); pre: mean (96.43), SD (23.16); post: mean (91.70), SD (23.71)  **-Nutrition**, assessed by energy (kj/24 hrs); pre: median (6904), interquartile range (5865-8054); post: median (7152), interquartile range (6107-8376) | Anthropometric assessments:  -**BMI Z score:** pre-mean (0.15), SD (1.2), post-mean (0.23), SD (1.27)  Behavioural assessments:  -**PA**: pre: mean (94.08), SD (24.38); post: mean (91.27), SD (25.42)  -**Nutrition**: pre: median (6911), interquartile range (5804-7964); post: median (7074), interquartile range (5963-8233) | This experiential focused intervention had no statistically significant effect on BMI z score or on preventing childhood obesity. | the National Institute for Health  Research (NIHR) Health Technology Assessment Programme (project  reference No 06/85/11) |
| Pablos A, 2017 | Cluster RCT | **Country**: Spain; **Attrition rate**: NA; **Age**: mean (10.66), SD (0.712); **Female (%)**: 51.9; **Sample size**: 4 schools, 158 children | **2 schools, 82 children**  **Theory:** NA;  **Intervention setting:** school & home;  **Content and frequency:** themed physical games, 2 sessions/wk, a total of 150 min/wk;  **Length:** 8 months  **Follow-up duration:** 8 months;  **Target population:** children, teachers and parents;  **Process evaluation:** NA | **2 schools, 76 children**  **Usual practice** | Anthropometric assessments:  -**BMI**: pre: mean (20.8), SD (4.7); post: mean (20.9), SD (4.6).  Behavioral assessments  **-PA:** assessed by self-reported questionnaire (Inventory of Healthy Habits); PA (min/wk)-pre: mean (436.4), SD (325); post: mean (508.5), SD (392)  **-Nutrition:** assessed by self-reported questionnaire (Inventory of Healthy Habits); food quality: pre-mean (57.4), SD (8.2), post-mean (58.6), SD (8.2) | Anthropometric assessments:  -**BMI**: pre: mean (19.6), SD (3.6); post: mean (20.3), SD (3.7)  Behavioral assessments  **-PA:** pre: mean (574.1), SD (365.77); post: mean (492.8), SD (367.2)  **-Nutrition:** pre-mean (56.8), SD (9.6); post-mean (53.6), SD (10.6) | The Healthy Habits Program is innovative and useful that can help to improve diet and health in childhood. | Universidad Católica de Valencia “San Vicente Martir” (Grant No.  2013/158/002) |
| ***PA+DI+/-SP*** |  |  |  |  |  |  |  |  |
| Foster, 2010[30] | Cluster RCT | **Country**: USA; **Attrition rate**: 1755 (27.6%); **Age**: mean (11.3 (0.6)); **Female (%)**: 52.7; **Sample size**: 42 schools, 4603 students | **21 schools, 2307 students  Theory:** the ecological model; **Intervention setting:** school mainly; **Content and frequency:** 1) nutrition (the quantity and nutritional quality of foods and beverages that were served throughout the school environment), 2) PA (increasing the amount of time spent in MVPA), 3) behavioral knowledge and skills (self-monitoring and goal setting), 4) communications and social marketing; **Length:** 2 school years; **Follow-up duration:** 2 school years; **Target population:** food service staff, students & teachers; **Process evaluation:** evaluated through structured observations. | **21 schools, 2296 students** No 'placebo' intervention was delivered. | Anthropometric assessments -**BMI Z:** pre (0.90 (1.08), post (0.85 (1.03)) | Anthropometric assessments -**BMI Z:** pre (0.87 (1.12), post (0.86 (1.05)) | The mean BMI Z score was sig. lower in the intervention schools than in the control schools. | the National Institutes of Health and the American Diabetes Association |
| Angelopoulos, 2009[31] | Cluster RCT | **Country**: Greece; **Attrition rate**: 0; **Age**: mean (10.3 (0.4)); **Female (%)**: 47.6; **Sample size**: 26 schools, 646 students | **13 schools, 321 students  Theory:** the Theory of Planned Behavior; **Intervention setting:** school & home; **Content and frequency:** overcoming the barriers in accessing PA areas, increasing the availability of fruits & vegetables and increasing parental support; **Length:** 12 mons; **Follow-up duration:** 12 mons; **Target population:** students & parents; **Process evaluation:** NA. | **13 schools, 325 students** Not receiving the intervention | Anthropometric assessments -**BMI:** pre (20.3 (3.6)), post (19.2 (2.9)); -**BMI Z:** pre (0.87 (0.9)), post (0.41 (0.9))  Behavioral assessments -**PA:** assessed using a standardized questionnaire completed by the children; MVPA (min/d): pre (41.1 (36.6)), post (43.4 (27.2)) -**Nutrition**: dietary intake was collected by the 24-hr recall technique for two consecutive weekdays and one weekend day; sig. differences were observed in the daily consumption of fruit, dairy, fats and oils, sweets and beverages. | Anthropometric assessments -**BMI:** pre (20.1 (3.4)), post (20.2 (3.2)); -**BMI Z:** pre (0.83 (0.9)), post (0.67 (0.8))  Behavioral assessments -**PA:**  pre (47.7 (41.9)), post (31.3 (23.6)) -**Nutrition**: has been compared in the left column | This 1-year school-based intervention program was effective in promoting favorable changes in BMI and obesity-related behaviors. | NA |
| Barbeau, 2007[32] | Individual RCT | **Country**: America; **Attrition rate**: 77 (27.7%); **Age**: mean (9.5), range (8-12); **Female (%)**: 100; **Sample size**: 8 schools, 278 students | **118 students analyzed  Theory:** NA; **Intervention setting:** school only; **Content and frequency:** 30-min homework/healthy snack time and 80-min PA/d after school; **Length:** 10 mons; **Follow-up duration:** 10 mons; **Target population:** students; **Process evaluation:** mean attendance was 54%. | **83 students analyzed**  Receiving no intervention | Anthropometric assessments -**BMI:** pre (20.9 (5.0)), post (21.6 (5.2))  Behavioral assessments -**PA:** measured using a 7-d recall; MVPA (hr/d): pre (0.46 (0.48), post (1.00 (0.67)) | Anthropometric assessments -**BMI:** pre (20.9 (5.6)), post (22.2 (6.1))  Behavioral assessments -**PA:** pre (0.46 (0.44)), post (0.67 (0.61)) | The after-school PA program can lead to beneficial changes in BMI and PA. | the NIH (Grant HL64972) |
| ***HE+DI+/-SP*** |  |  |  |  |  |  |  |  |
| Lloyd, 2012[33] | Cluster RCT | **Country**: England; **Attrition rate**: 9 (4.5%) at 18 mons, 15 (7.4%) at 24 mons; **Age**: mean (9.7 (0.3)), range (9-10); **Female (%)**: 50; **Sample size**: 4 schools, 202 students | **2 schools, 80 students   Theory:** behavior change techniques; **Intervention setting:** school & home; **Content and frequency:** lessons, assemblies, parents' evenings, interactive drama workshops, goal settings; **Length:** 12 mons; **Follow-up duration:** 24 mons; **Target population:** students, teachers & parents; **Process evaluation:** NA. | **2 schools, 122 students**  Usual practice | Anthropometric assessments -**BMI:** adjusted mean difference at 18 mons (-0.95 (-3.79 to 1.90)) **-BMI Z:** adjusted mean difference at 18 mons (-0.38 (-1.65 to 0.89))  Behavioral assessments -**PA:** assessed by accelerometers; MVPA (mins/d): adjusted mean difference at 18 mons (5.67 (-12.59 to 23.93)) **-Nutrition:** at 18 mons of follow-up, children in the intervention schools had less 'negative food markers', consumed less energy-dense food and more health food, had more 'positive food markers'. | Has been compared with the intervention group in the left column | This exploratory trial show consistent positive changes in favor of the intervention across behaviors, which appear to affect weight status. | The National Institute for Health Research (NIHR) Research for Patient Benefit Program |
| Xu, 2015[34] | Cluster RCT | **Country**: mainland China; **Attrition rate**: 74(6.3%); **Age**: mean (10.2 (0.5)); **Female (%)**: 47.7; **Sample size**: 8 schools, 1108 students | **4 schools, 635 students   Theory:** the Theory of Triadic Influence, the Comprehensive School Health Program Model; **Intervention setting:** school & home; **Content and frequency:** 1) classroom curriculum (1 30-min lesson/mon, 2) school environment support, 3) family involvement, 4) fun programs/events; **Length:** 1 academic school year (8 mons); **Follow-up duration:** 8 mons; **Target population:** students, teachers & parents; **Process evaluation:** NA. | **4 schools, 503 students**  Routine health education | Anthropometric assessments -**BMI:** pre (18.71 (3.17)), post (18.39)  Behavioral assessments -**PA:** compared with the control group, the intervention group was more likely to increase the frequency of jogging/running (OR=1.55, 95%CI=1.18, 2.02） **-Nutrition:** compared with the control group, the intervention group was more likely to reduce red meat consumption (OR=1.50, 95%CI=1.15, 1.95) | Anthropometric assessments -**BMI:** pre (18.54 (2.92)), post (18.25)  Behavioral assessments -has been compared with the intervention group in the left column | The school-based lifestyle intervention was effective in improving health behaviors. | The study was supported by the Nanjing Municipal Science and Technique Foundation (200901088), Medical Science and Technique Development Foundation (ZKX09034) and The Young Medical Experts Project of Nanjing Medical Science and technique Development Foundation (QRX11038) and Nanjing Municipal Center for Disease Control and Prevention (Nanjing CDC), China. |
| Kipping, 2014[35] [49] | Cluster RCT | **Country**: UK; **Attrition rate**: 17 (0.9%) for BMI measure; **Age**: mean (9.5 (0.3)), range (9-10); **Female (%)**: 50.8; **Sample size**: 60 schools, 2221 students | **30 schools, 1064 students   Theory:** social cognitive theory, a particular emphasis on improving children's self-efficacy; **Intervention setting:** school & home; **Content and frequency:** 1) teacher training (8-9 hrs), 2) provision of classes (16 lessons), 3) 10 child-parent interactive homeworks; **Length:** 7 mons; **Follow-up duration:** 7 mons; **Target population:** students, teachers & parents; **Process evaluation:** fidelity of intervention was good. | **30 schools, 1157 students**   Standard teaching | Anthropometric assessments -**BMI Z:** adjusted mean differences between two groups~-0.02 (-0.08 to 0.03)  Behavioral assessments -**PA:** assessed by accelerometer; adjusted mean differences between two groups: -1.35 (-5.29 to 2.59) -**Nutrition:** self-reported daily consumption of servings of fruit and vegetables; adjusted mean differences between two groups: 0.08 (-0.12 to 0.28) | Has been compared in the left column | The school-based intervention is not effective at increasing PA or increasing fruit and vegetable consumption in primary school children. | Main funding: the UK National Institute for Health Research (NIHR) Public Health Research Programme (09/3005/04) |
| Scherr, 2017[36] | Cluster RCT | **Country**: America; **Attrition rate**: NA; **Age**: mean (9.6), range (9-10); **Female (%)**: 49.7; **Sample size**: 4 schools, 443 students | **2 schools, 245 students   Theory:** Social Cognitive Theory, Social Ecological Model; **Intervention setting:** school, home & community; **Content and frequency:** 1) nutrition education and promotion (15 classroon lessons & 19 take-home activities), 2) family and community partnership, 3) supporting regional agriculture, 4) foods available on the school campus, 5) school wellness and policies; **Length:** one school year; **Follow-up duration:** one school year; **Target population:** students, parents, teachers & community partners; **Process evaluation:** degree of program completion was assessed. | **2 schools, 198 students**  "The nutrition educator spent time in the control classrooms to match the number of hours spent in the intervention classrooms to mitigate the potential of the Hawthorne effect. …The content was unrelated to nutrition, health, and science." | Anthropometric assessments -**BMI Z:** pre (0.81 (1.05)), post (0.53 (1.04))  Behavioral assessments **-Nutrition:** assessed by food frequency questionnaire; reported fruit intake (servings) (pre: 1.84 (1.36), post: 1.76 (1.43)); reported vegetable intake (pre: 0.91 (0.86), post: 0.81 (0.79)) | Anthropometric assessments -**BMI Z:** pre (0.48 (1.08)), post (0.41 (1.10))  Behavioral assessments -**Nutrition:** reported fruit intake (pre: 2.02 (1.40), post: 1.87 (1.42)); reported vegetable intake (pre: 0.74 (0.51), post: 0.75 (0.60)) | The intervention resulted in a greater improvement in BMI Z-score. | University of California Agriculture and Natural Resources Competitive Grant 11-1018, US Department of Agriculture Nutrition Institute of Food and Agriculture HATCH Project 221082, andUSDepartmentofAgricultureTraining Grant 2011-38420-20082, and University of California Supplemental Nutrition Assistance Program–Education (SNAP-Ed) Funds |
| Habib-Mourad, 2014[37] | Cluster RCT | **Country**: Lebanon; **Attrition rate**: 11 (2.9%); **Age**: mean (10), range (9-11); **Female (%)**: 44.1; **Sample size**: 8 schools, 374 students | **4 schools, 193 students   Theory:** Social Cognitive Theory; **Intervention setting:** school & home; **Content and frequency:** 1) class curriculum (12 45-min sessions), 2) family involvement, 3) food service; **Length:** 3 mons; **Follow-up duration:** 3 mons; **Target population:** students, teachers & parents; **Process evaluation:** the programme was delivered as designed, but the implementation of the DI component was not reported. | **4 schools, 181 students** Receiving usual school curriculum | Anthropometric assessments -**BMI:** pre (19.7 (4.0)), post (20.2 (4.2))   Behavioral assessments, assessed based on self-report measures: **-PA:** no difference between groups; -**Nutrition**: compared with the control group, daily breakfast intake increased sig., consumed less chips and sweetened drinks | Anthropometric assessments -**BMI:** pre (18.8 (3.5)), post (19.0 (3.5))   Behavioral assessments **-**has been compared with the intervention group in the left column | The intervention is beneficial in promoting healthy eating and PA. | the Eastern Mediterranean Regional Office Special Grant for Research in Priority Areas of Public Health (EMRPPH) |
| Foster, 2008[38] | Cluster RCT | **Country**: America; **Attrition rate**: 428 (31.7%) at year 1 505 (37.4%) at year 2; **Age**: mean (11); **Female (%)**: 53.7; **Sample size**: 10 schools, 1349 students | **5 schools, 749 students   Theory:** NA; **Intervention setting:** school & home; **Content and frequency:** 1) school self-assessment, 2) nutrition education (~10 hrs/year for school staff; 50 hrs/year for school students), policy, 3) social marketing and parent outreach; **Length:** 2 years; **Follow-up duration:** 2 years; **Target population:** students, teachers & parents; **Process evaluation:** NA. | **5 schools, 600 students** no changes were made | Anthropometric assessments -**BMI:** pre (20.98 (5.1)), post (23.06) **-BMI Z:** pre (0.71 (1.1)), post (0.80)  Behavioral assessments, assessed based on self-report measures: **-PA:** decreases in PA were similar between two groups; -**Nutrition**: decreases in consumption of energy, fat, fruits and vegetable were similar between two groups. | Anthropometric assessments -**BMI:** pre (20.71 (5.0)), post (22.86)  **-BMI Z:** pre (0.65 (1.1)), post (0.76)  Behavioral assessments, has been compared in the left column | The intervention had no effect on BMI indices, but did be effective in preventing the development of overweight. | the US Department of Agriculture Food Stamp Nutrition Education Program |
| Rush, 2012[39] | Cluster RCT | **Country**: New Zealand; **Attrition rate**: 20% of the younger children and 43% of the older children; **Age**: range (5-10); **Female (%)**: 49.3; **Sample size**: 124 schools, 1352 students | **62 schools, 692 students   Theory:** NA; **Intervention setting:** school; **Content and frequency:** 1) "Energizer" (a trained PA and nutrition change agent)-led physical activity sessions, 2) assistance with a range of health eating initiatives, 3) offering assistance to teachers, parents and local community; **Length:** 23 mons; **Follow-up duration:** 23 mons; **Target population:** students, teachers, parents & the local community; **Process evaluation:** NA. | **62 schools, 660 students** Being "given no additional resourcing or information; no restrictions were placed on initiative they may have pursed for themselves" | Anthropometric assessments **-BMI Z,** adjusted mean differences between two groups after the 2-year intervention: 1) for 5-7-year-old: 0.00 (-0.06, 0.06) 2) for 10-12-year-old: 0.05 (-0.04, 0.13) | Has been compared with the intervention group in the left column | The introduction of an 'Energizer led" through school program might be associated with health benefits over a longer period. | The Waikato District Health Board funds the Project Energize programme and its evaluation. The Ministry of Health, New Zealand has contributed to evaluation funding. |
| ***PA+HE+DI+/-SP*** |  |  |  |  |  |  |  |  |
| Safdie, 2013[40] | Cluster RCT | **Country**: Mexico; **Attrition rate**: for schools: 1 plus school was excluded at the 2nd year; for students: 56 (6.3%); **Age**: mean (9.7 (0.7)); **Female (%)**: 51.5; **Sample size**: 27 schools, 886 students | **Intervention 1 (Basic intervention group): 8 schools, 262 students Intervention 2 (Plus intervention group): 8 schools, 264 students   Theory:** ecological principles, the theory of planned behavior, social cognitive theory, health belief model; **Intervention setting:** school; **Content and frequency:**   1) Intervention 1: nutrition+PA;  2) Intervention 2: all components in Intervention 1 + 1 PE class/week + (15-20) minutes of MVPA (4 d/wk) **Length:** 18 mons; **Follow-up duration:** 18 mons; **Target population:** students & teachers; **Process evaluation:** the nutrition component was more successful, while the PA component was less successful (over 30% of the scheduled PE classes were canceled). | **11 schools, 360 students** No changes were made to existing nutrition or PA practices | Anthropometric assessments **-BMI**  1) Intervention 1: pre 19.4 (19.0-19.9), post 20.4 (19.6-21.2)  2) Intervention 2: pre 20.0 (19.5-20.5), post 19.0 (18.2-19.9)  Behavioral assessments **-PA**   1) PA opportunities during PE classes and recess, assessed by recording students' levels of PA using SOFIT (System for Observing Fitness Instruction Time): differences were no sig. between groups  2) steps taken at school were measured by pedometers: increased in intervention while decreased in control **-Nutrition** 1) food and beverage availability at school, assessed using food inventories, completed by nutritionists: compared with the control group, there was an increase in the percentages of highly recommended food items, the availability of potable drinking water, and a reduction in the percentage of non-recommended food items in the intervention group;  2) food intake at recess, observed and recorded by the field personnel: compared with the control group, children's intake of highly recommended and recommended for consumption increased in intervention, while intake of non-recommended foods decreased in intervention | Anthropometric assessments -**BMI**  pre 19.9 (19.5-20.3), post 19.1 (18.4-19.8)  Behavioral assessments, has been compared with the intervention group in the left column | The intervention improved the school food environment and child healthy behaviors. | the Global Health Research Initiative (GHRI) |
| Gallotta, 2016[41] | Cluster RCT | **Country**: Italy; **Attrition rate**: 0; **Age**: range (8-11); **Female (%)**: 43.5; **Sample size**: 3 schools, 230 students | **1. Intervention1, 78 students  2. Intervention2, 83 students  Theory:** NA; **Intervention setting:** school; **Content and frequency:**  1) Intervention1: PA component (conducted by a specialist PE teacher and structured) + Nutritional component   2) Intervention2: PA component (conducted by a specialist PE teacher and focused on high varied activities) + Nutritional component **Length:** 5 mons; **Follow-up duration:** 5 mons; **Target population:** students, parents & teachers; **Process evaluation:** NA. | 69 students  Traditional PE school curriculum (conducted by the generalist teacher and structured) | Anthropometric assessments **-BMI Z (1) Intervention1**  1) for under fat children: pre -0.14 (0.92), post 0.13 (1.01)  2) for normal fat children: pre 0.77 (0.73), post 0.68 (0.69)  3) for overweight/obese children: pre 2.01 (0.32), post 1.96 (0.31) **(2) Intervention2**  1) for under fat children: pre -0.7 (1.01), post -0.58 (0.84)  2) for normal fat children: pre 0.63 (0.78), post 0.72 (0.65)  3) for overweight/obese children: pre 1.99 (0.27), post 1.97 (0.30)  Behavioral assessments, assessed by self-reported questionnaire **-PA:** the control group had lower PA level than Intervention1 and Intervention2 **-Nutrition:** the effects of group on changes of some foods (potatoes, fish, eggs, cold cuts, sweets, drinks, snacks, fruit) consumption was presented. | Anthropometric assessments **-BMI Z**  1) for under fat children: pre -0.17 (0.75), post -0.12 (0.76)  2) for normal fat children: pre 0.33 (0.97), post 0.43 (0.94)  3) for overweight/obese children: pre 2.13 (0.39), post 2.10 (0.40)  Behavioral assessments **-PA**  **-Nutrition** has been compared with the intervention group in the left column | The combined PE and nutritional intervention effectively improved children's diet and PA levels. | Department of Movement, Human and Health Sciences: year 2014 Cod. RIC062014. University of Rome ``Foro Italico,'' Rome, Italy: PRIN 2009 2009KENS9K_004. |
| Kain, 2014[42] | Cluster RCT | **Country**: Chile; **Attrition rate**: 475 (24.4%); **Age**: mean (6.6 (1.1)); **Female (%)**: 46.6; **Sample size**: 9 schools, 1949 students | **5 schools, 651 students   Theory:** NA; **Intervention setting:** school; **Content and frequency:** 1) classroom education on nutrition, 2) improve the quality of PE classes; **Length:** 12 mons; **Follow-up duration:** 12 mons; **Target population:** students, teachers & parents; **Process evaluation:** ~80% of the nutrition contents were implemented. | **4 schools, 823 students** | Anthropometric assessments **-BMI Z：** pre, means of 5 schools ranged from 0.77 to 1.4;  post, means of 5 schools ranged from 0.91 to 1.31  Behavioral assessments, assessed by self-reported questionnaire **-PA, assessed by pedometers (MVPA, mins/PE class):** pre 15.6 (5.3), post 16.1 (5.0) | Anthropometric assessments **-BMI Z：** pre, means of 4 schools ranged from 0.99 to 1.22;  post, means of 4 schools ranged from 1.1 to 1.39    Behavioral assessments, assessed by self-reported questionnaire **-PA:** pre 13.8 (5.6), post 10.2 (5.6) | The intervention was effective in controlling obesity, but not preventing it. | the “Corporaci´on Municipal de Educaci´on y Salud” of ˜ Nu˜noa |
| Caballero, 2003[43] | Cluster RCT | **Country**: America; **Attrition rate**: 295 (17.3%); **Age**: mean (7.6 (0.6)); **Female (%)**: NA; **Sample size**: 41 schools, 1704 students | **879 students   Theory:** social learning theory; **Intervention setting:** school & home; **Content and frequency:** 1.change in dietary intake 2.increase in physical activity (3 30-min PE sessons/wk) 3.classroom curriculum focused on healthy eating and lifestyle (12wk/y (8 wk in the 5th grade), twice weekly for 45-min as a lesson) 4. with the involvement of family; **Length:** 3 years; **Follow-up duration:** 3 years; **Target population:** students, teachers, food service staff & parents; **Process evaluation:** 1) 94% of classroom curriculum was completed, 2) 3 PE sessions/wk was achieved by 81% of schools in the 1st year, and by 100% in years 2 and 3, 3) 58% attended family events, 4) fat intake was lower in the lunch of intervention school. | **825 students** Not clearly reported | Anthropometric assessments -**BMI**: pre (19.0), post (22.0)  Behavioral assessments -**PA**: assessed by both motion sensor & questionnaire  1) motion sensor (average vector magnitude/min): pre 282.04, post 267.22  2) questionnaire: pre 0.35, post 0.27 -**Nutrition**: assessed by school-lunch observation  1) energy (kcal): pre 522.9, post 500.2  2) Fat (% of energy): pre 33.1, post 28.2 | Anthropometric assessments -**BMI**: pre (19.1), post (22.2)  Behavioral assessments -**PA**:  1) motion sensor: pre 303.13, post 246.79  2) questionnaire: pre 0.35, post 0.24 -**Nutrition**:  1) energy: pre 573.6, post 494.4  2) Fat: pre 34.1, post 32.4 | The program produced positive changes in fat intake, but to reduce adiposity, more intense or longer interventions may be needed. | National Heart, Lung, and Blood Institute grants U01- HL-50869, -50867, -50905, -50885, and -50907 |
| Singhal, 2010[44] | Cluster RCT | **Country**: India; **Attrition rate**: 8 (3.8%); **Age**: mean (16.0 (0.5)), range (15-17); **Female (%)**: 39.8; **Sample size**: 2 schools, 201 students | **1 school, 99 students   Theory:** NA; **Intervention setting:** school; **Content and frequency:** 1) lectures & focus group discussions (24 wks), 2) promotion of PA, 3) health promotion activities (8 wks), 4) individual counseling (an hr every wk), 5) policy-level changes in school (changing the school canteen menu), 6) involvement of teachers and parents (for both, 1-d meeting; for parents, 5-7 min/mon), 7) training of student volunteers (1-h/wk). **Length:** 8 mons; **Follow-up duration:** 8 mons; **Target population:** students, teachers & parents; **Process evaluation:** NA. | **1 school, 102 students** Not receiving any intervention | Anthropometric assessments -**BMI**: change from baseline to follow-up (-0.07 (0.71))  Behavioral assessments, assessed by questionnaire -**PA**: compared with the control group, higher proportion of children engaged in PA at least 4d/week for at least 30-60 min; -**Nutrition**: compared with the control school, consumption of milk, fresh fruits increased, and consumption of white bread, aerated drinks and energy-dense unhealthy foods decreased | Anthropometric assessments -**BMI**: change from baseline to follow-up (-0.06 (1.11))  Behavioral assessments -has been compared with the intervention group in the left column | The multi-component intervention was successful in improving behaviors and resulted in beneficial changes in anthropometric profiles. | The World Diabetes Foundation, Denmark (WDF05-120) |
| Singh, 2007[45] | Cluster RCT | **Country**: Netherland; **Attrition rate**: 75 (7.1%); **Age**: mean (12.7 (0.5)); **Female (%)**: 53.3; **Sample size**: 18 schools, 1053 students | **10 schools, 600 students   Theory:** ANalysis Grid for Environments Linked to Obesity (ANGELO); **Intervention setting:** school; **Content and frequency:** an adapted curriculum for 11 biology and PE lessons and environmental change options, including additional lessons on PE and advice on the school canteen selections; **Length:** 8 mons; **Follow-up duration:** 8 mons; **Target population:** students & teachers; **Process evaluation:** NA. | **8 schools, 453 students**  Maintaining regular curriculum | Anthropometric assessments -**BMI**: pre (19.0 (3.0)), post (19.5 (3.1)) | Anthropometric assessments -**BMI**: pre (19.5 (3.4)), post (20.0 (3.5)) | The intervention had positively influenced anthropometric measures. | This study is part of Netherlands Research Programme Weight Gain Prevention and is funded by grant 2000Z003 from the Netherlands Heart Foundation and by the Dutch Ministry of Health, Welfare, and Sports and the Royal Association of Teachers of Physical Education |
| Grydeland, 2013[46] [50] | Cluster RCT | **Country**: Norway; **Attrition rate**: 219 (13.9%); **Age**: mean (11.2 (0.3)), range (11-13); **Female (%)**: 48.6; **Sample size**: 37 schools, 1324 students | **12 schools, 566 students   Theory:** social ecological frameworks, dual-process model, social cognitive theories, the analysis grid for environments linked to obesity (ANGELO); **Intervention setting:** school; **Content and frequency:** 1) class-based activities (monthly lessons, monthly posters, weekly 'fruit and vegetable break', weekly 10-min PA break, sports equipment, 5 active commuting campaigns for 3 wks, pedometer used, computer tailored individual advice on PA and eating plus 1-wk action plan); 2) home/parents: provision of fact sheets and brochures; 3) school-wide: yearly meetings for 'HEIA team' teachers, yearly courses for PE teachers; 4) leisure time activities. **Length:** 20 mons; **Follow-up duration:** 20 mons; **Target population:** students, teachers & parents; **Process evaluation:** NA. | **25 schools, 1014 students** Control | Anthropometric assessments -**BMI**: pre (17.8 (2.5), post (18.8 (18.7 to 18.9)  Behavioral assessments -**PA** assessed by accelerometer (counts/min):   1) estimated PA at school: pre (604 (188)), post (559 (208))  2) estimated PA after school: pre (432 (217)), post (622 (421)) | Anthropometric assessments -**BMI**: pre (17.9 (2.6), post (18.9 (18.8 to 18.9))  Behavioral assessments -**PA:** 1) estimated PA at school: pre (621 (189)), post (582 (223))  2) estimated PA after school: pre (504 (248)), post (599 (381)) | 1. The multi-component intervention can affect PA patterns in adolescents, and the effect was more profound in girls. 2. The intervention had a beneficial effect on BMI indices and the effect was more profound in girls. | Funded by the Norwegian Research Council (grant number 175323/V50) with supplementary funds from the Throne Holst Nutrition Research Foundation, University of Oslo and the Norwegian School of Sport Science |
| Liu 2019 | Cluster RCT | **Country**: mainland China; **Attrition rate**: 50 (2.6); **Age**: range (7-11); **Female (%)**: 48.3; **Sample size**: 12 schools, 1889 students | **6 schools, 930 students   Theory:** the analysis grid for environments linked to obesity (ANGELO); **Intervention setting:** school & home; **Content and frequency:** 1) school-level policies 2) health education activities 3) improvement of physical activity 4) improvement of school lunches; **Length:** 12 mons; **Follow-up duration:** 12 mons; **Target population:** students, teachers & parents; **Process evaluation:** via researchers’ records of field work and direct observation. | **6 schools, 959 students** Control | Anthropometric assessments -**BMI**: pre 17.84 (3.37), post 18.57 (3.60)  -**BMI Z:** pre 0.30 (1.20), post 0.31 (1.19)  Behavioral assessments **-PA:** assessed by self-reported questionnaire (number of days doing ≥ 1 hr MVPA/week); pre 2 (0-5), post 3.94 (2.52) | Anthropometric assessments -**BMI**: pre 17.85 (3.55), post 18.46 (3.76)  -**BMI Z:** pre 0.29 (1.23), post 0.28 (1.23)  Behavioral assessments **-PA:** pre 2(0-5), post 2.55 (2.46) | The intervention did not mitigate excess weight gain, but did improve children’s knowledge and several behaviors related to energy balance | China Medical Board (Project No. 11-064) |
| RCT: randomized controlled trial; BMI: body mass index; PA: physical activity; HE: health education; DI: dietary improvement; SP: school policy; NA: not appropriate as the original study did not provide this data; PE: physical education; mon: month; wk: week; d: day; hr: hour; min: minute; MVPA: moderate-to-vigorous physical activity; LPA: low-intensity physical activity; MPA: moderate-intensity physical activity; VPA: vigorous physical activity; pre: pre-intervention; post: post-intervention; sig.: significant/significance; CI: confidence interval; OR: odds ratio; The figures in the brackets mean the standard deviations/percentages for the means/numbers before the brackets. | | | | | | | | |

| **Table S2 Description of the characteristics of the PA component for the included studies** | | | |
| --- | --- | --- | --- |
| **Author, year** | **PA** $\boldsymbol{\geq}$**3 times/week &** $\boldsymbol{\geq}$**10min/time** | **Having curricular PA** | **PA’s enjoyment** |
| ***PA*** |  |  |  |
| Drummy, 2016[1] | $\times$ | $\times$ | $\times$ |
| Kriemler, 2010[2] | $\surd$ | $\surd$ | $\surd$ |
| Thivel, 2011[3] | $\times$ | $\surd$ | $\surd$ |
| Aguilar, 2009[4] | $\surd$ | $\times$ | $\surd$ |
| Donnelly, 2009[5] | $\surd$ | $\times$ | $\times$ |
| Li, 2010[6] | $\surd$ | $\times$ | $\surd$ |
| Sacchetti, 2013[7] | $\surd$ | $\surd$ | $\surd$ |
| ***PA+SP*** |  |  |  |
| Hollis, 2016[16] | ? | $\surd$ | $\times$ |
| Ansari, 2010[17] | $\surd$ | $\times$ | $\surd$ |
| ***PA+HE+/-SP*** |  |  |  |
| Jansen, 2011[22] | $\surd$ | $\surd$ | $\times$ |
| Manios, 2002[23] | $\times$ | $\surd$ | $\surd$ |
| Peralta, 2009[24] | $\surd$ | $\surd$ | $\surd$ |
| Vandongen, 1995[25] | $\surd$ | $\times$ | $\times$ |
| Siegrist, 2011[26] | $\times$ | $\surd$ | $\times$ |
| Jiang, 2006[27] | $\surd$ | $\surd$ | $\times$ |
| Magnuson, 2012[28] | $\surd$ | $\surd$ | $\times$ |
| Dewar, 2013[29] | $\times$ | $\times$ | $\surd$ |
| Adab, 2018 | $\surd$ | $\surd$ | $\times$ |
| Pablos, 2017 | $\times$ | $\surd$ | $\surd$ |
| ***PA+DI+/-SP*** |  |  |  |
| Foster, 2010[30] | ? | $\surd$ | ? |
| Angelopoulos, 2009[31] | $\times$ | $\surd$ | $\surd$ |
| Barbeau, 2007[32] | $\surd$ | $\times$ | $\surd$ |
| ***PA+HE+DI+/-SP*** |  |  |  |
| Safdie, 2013[40] | $\surd$ | $\surd$ | $\times$ |
| Gallotta, 2016[41] | ? | $\surd$ | ? |
| Kain, 2014[42] | $\times$ | $\surd$ | $\times$ |
| Caballero, 2003[43] | $\surd$ | $\surd$ | $\times$ |
| Singhal, 2010[44] | $\times$ | $\times$ | $\times$ |
| Singh, 2007[45] | $\times$ | $\surd$ | $\times$ |
| Grydeland, 2013[46] | ? | $\times$ | ? |
| Liu 2019 | $\surd$ | $\surd$ | $\surd$ |
| PA: physical activity; HE: health education; DI: dietary improvement; SP: school policy; “?”: unclear as detailed information was not provided in the original report; “$\surd$”: the characteristics of the PA component was included in the study; “$\times$”: the characteristics of the PA component was not included in the study. | | | |

| **Table S3 Description of the characteristics of the DI component for the included studies** | |
| --- | --- |
| **Author, year** | **Description of the DI component** |
| ***PA+DI+/-SP*** |  |
| Foster, 2010[30] | Targeting the quantity and nutritional quality of foods and beverages that were served throughout the school environment |
| Angelopoulos, 2009[31] | Increasing the availability of fruits and vegetables at school and at home |
| Barbeau, 2007[32] | Provision with a healthy snack free of charge |
| ***HE+DI+/-SP*** |  |
| Lloyd, 2012[33] | Targeted information, motivation and behavioral skills related to DI |
| Xu, 2015[34] | Mainly through health education |
| Kipping, 2014[35] | Mainly through health education |
| Scherr, 2017[36] | Mainly through health education and modification of school environment |
| Habib-Mourad, 2014[37] | Increasing the availability of healthy food as well as decreasing the availability of unhealthy food at school based on the voluntary principle |
| Foster, 2008[38] | Through both nutrition education and policy on a mandatory basis |
| Rush, 2012[39] | Healthy-eating initiatives (removing unhealthy food in canteen, raising healthy fund) |
| ***PA+HE+DI+/-SP*** |  |
| Safdie, 2013[40] | Increasing the availability of healthy food and water, reducing the availability of energy-dense foods and sugar-sweetened beverages, reducing the number of eating opportunities during the school day |
| Gallotta, 2016[41] | Health education; provision with 1 free piece of fresh fruit/vegetable weekly |
| Kain, 2014[42] | Mainly through health education |
| Caballero, 2003[43] | Food service in school meals (reducing the fat content) |
| Singhal, 2010[44] | Policy-level changes in school (changing the school canteen menu) |
| Singh, 2007[45] | Advice on the school canteen selections |
| Grydeland, 2013[46] | Weekly ‘fruit and vegetable break’ |
| Liu, 2019 | Improvement of school lunches |

DI: dietary improvement

**Table S4 Differences of overall effect size by sex, weight status and country of the study population**

| **Characteristics of the study population** | **No. of studies** | **Qualitative summary** | **Quantitative summary** |
| --- | --- | --- | --- |
| **Sex** | - 19 studies reported differences of overall effect size by sex. - 8 studies reported sub-group data by sex. | - 16 studies (84.2%) reported that intervention effects were similar between boys and girls. - 3 studies (15.8%) reported that girls responded better than boys to the same intervention. | - **Boys:** -0.09 (-0.38, 0.20) in BMI, 0.14 (-0.15, 0.43) in BMI Z-score; - **Girls:** -0.29 (-0.50, -0.08) in BMI, -0.12 (-0.23, -0.00) in BMI Z-score. - **Tests for subgroup differences:** *P*=0.27 for BMI**;** *P*=0.11 for BMI Z-score. |
| **Weight status at baseline** | - 7 studies reported differences of overall effect size by weight status at baseline. - 3 studies reported sub-group data by weight status at baseline. | - 6 studies (85.7%) reported that intervention effects were similar across weight status at baseline. - 1 study (14.3%) reported that the intervention had a more pronounced effect among obese children than among normal weight or overweight children. | - **Children without overweight or obese:** -0.15 (-0.26, -0.04) in BMI, -0.07 (-0.13, -0.01) in BMI Z-score; - **Children with overweight or obese:** -0.08 (-0.13, -0.02) in BMI, -0.05 (-0.09, -0.01) in BMI Z-score; - **Tests for subgroup differences:** *P*=0.23 for BMI; *P*=0.57 for BMI Z-score. |
| **Country** | A total of 50 studies   - 10 studies were conducted in middle-income countries; - 40 studies were conducted in high-income countries. | **-** | - **Interventions conducted in middle-income countries:** -0.56 (-1.00, -0.12) in BMI, -0.06 (-0.12, 0.00) in BMI Z-score; - **Interventions conducted in high-income countries:** -0.16 (-0.36, 0.03) in BMI, -0.07 (-0.14, -0.01) in BMI Z-score. - **Tests for subgroup differences:** *P*=0.11 for BMI; *P*>0.05 for BMI Z-score. |

| **Table S5 Subgroup analyses by characteristics of multi-component interventions (excluding heterogeneous studies)** | | | | | | |
| --- | --- | --- | --- | --- | --- | --- |
| **Outcomes** | **BMI** | | | **BMI Z-score** | | |
|  | N | Mean difference, 95% CI | *P* for subgroup analyses | N | Mean difference, 95% CI | *P* for subgroup analyses |
| **Characteristics of the PA component** | | | | | | |
| **1) *PA’s frequency and duration*** |  |  |  |  |  |  |
| ≥3/week and ≥10 min/time | 8 | -0.26 (-0.74, 0.22) | 0.89 | - | - | - |
| <3/week or <10 min/time | 8 | -0.29 (-0.76, 0.19) |  | - | - |  |
| **2) *Curricular PA*** |  |  |  |  |  |  |
| Yes | 14 | -0.28 (-0.60, 0.05) | 0.83 | 5 | -0.09 (-0.22, 0.05) | 0.07 |
| No | 3 | -0.23 (-0.56, 0.11) |  | 2 | -0.48 (-1.17, 0.22) |  |
| **3) *PA emphasizing enjoyment*** |  |  |  |  |  |  |
| Yes | 7 | -0.42 (-0.98, 0.14) | 0.52 | - | - | - |
| No | 10 | -0.21 (-0.51, 0.10) |  | - | - | - |
| **Topics of HE covering both energy intake and output** |  |  |  |  |  |  |
| Yes | 15 | -0.28 (-0.57, 0.01) | 0.20 | 8 | -0.17 (-0.29, -0.04) | 0.18 |
| No | 7 | -0.07 (-0.20, 0.06) |  | 5 | -0.06 (-0.15, 0.03) |  |
| **Inclusion of the DI component** |  |  |  |  |  |  |
| Yes | 12 | -0.22 (-0.63, 0.18) | 0.75 | 10 | -0.07 (-0.18, 0.04) | 0.76 |
| No | 13 | -0.31 (-0.67, 0.04) |  | 6 | -0.05 (-0.07, -0.03) |  |
| **Inclusion of the SP component** |  |  |  |  |  |  |
| Yes | 17 | -0.21 (-0.48, 0.06) | 0.80 | 11 | -0.04 (-0.08, -0.01) | 0.52 |
| No | 6 | -0.17 (-0.33, -0.01) |  | 2 | -0.08 (-0.20, 0.03) |  |
| Abbreviations: CI: confidence interval; SP: school policy; PA: physical activity; HE: health education | | | | | | |

| **Table S6 Subgroup analyses by characteristics of single-component interventions (excluding trials assessed as high risk of bias)** | | | | | | |
| --- | --- | --- | --- | --- | --- | --- |
| **Outcomes** | **BMI** | | | **BMI Z-score** | | |
|  | N | Mean difference, 95% CI | *P* for subgroup analyses | N | Mean difference, 95% CI | *P* for subgroup analyses |
| **Characteristics of the PA component** |  |  |  |  |  |  |
| ***1) PA’s frequency and duration*** |  |  |  |  |  |  |
| ≥3/week and ≥10min/time | 4 | -0.14 (-0.26, -0.02) | 0.27 | - | - | - |
| <3/week or <10min/time | 2 | -0.31 (-0.57, -0.05) |  | - | - |  |
| ***2)* *Curricular PA*** |  |  |  |  |  |  |
| Yes | 3 | -0.30 (-0.51, -0.10) | 0.02 | - | - | - |
| No | 3 | -0.06 (-0.17, 0.05) |  | - | - |  |
| ***3)* *PA emphasizing enjoyment*** |  |  |  |  |  |  |
| Yes | 4 | -0.26 (-0.40, -0.12) | 0.004 | - | - | - |
| No | 2 | -0.004 (-0.10, 0.09) |  | - | - |  |
| **Topics of HE covering both energy intake and output** |  |  |  |  |  |  |
| Yes | 2 | -0.10 (-0.31, 0.12) | - | 4 | -0.07 (-0.19, 0.04) | - |
| No | 1 | -0.40 (-1.01, 0.21) |  | 1 | -0.12 (-0.28, 0.04) |  |
| CI: confidence interval; SP: school policy; PA: physical activity; HE: health education; DI: dietary improvement.  “--” due to insufficient observations. | | | | | | |

| **Table S7 Subgroup analyses by characteristics of multi-component interventions (excluding trials assessed as high risk of bias)** | | | | | | |
| --- | --- | --- | --- | --- | --- | --- |
| **Outcomes** | **BMI** | | | **BMI Z-score** | | |
|  | N | Mean difference, 95% CI | *P* for subgroup analyses | N | Mean difference, 95% CI | *P* for subgroup analyses |
| **Characteristics of the PA component** | | | | | | |
| **1) *PA’s frequency and duration*** |  |  |  |  |  |  |
| ≥3/week and ≥10min/time | 8 | -0.27 (-0.51, -0.04) | 0.96 | - | - | - |
| <3/week or <10min/time | 8 | -0.29 (-0.76, 0.19) |  | - | - |  |
| **2) *Curricular PA*** |  |  |  |  |  |  |
| Yes | 12 | -0.20 (-0. 59, 0.18) | 0.02 | 6 | -0.07 (-0.20, 0.06) | - |
| No | 4 | -1.29 (-2.15, -0.44) |  | 1 | -0.12 (-0.28, 0.04) |  |
| **3) *PA emphasizing enjoyment*** |  |  |  |  |  |  |
| Yes | 8 | -0.88 (-1.42, -0.34) | 0.006 | - | - | - |
| No | 8 | -0.11 (-0.20, -0.02) |  | - | - | - |
| **Topics of HE covering both energy intake and output** |  |  |  |  |  |  |
| Yes | 14 | -0.20 (-0.50, 0.09) | 0.42 | 7 | -0.19 (-0.33, -0.04) | 0.14 |
| No | 7 | -0.07 (-0.20, 0.06) |  | 5 | -0.06 (-0.15, 0.03) |  |
| **Inclusion of the DI component** |  |  |  |  |  |  |
| Yes | 12 | -0.22 (-0.63, 0.18) | 0.35 | 10 | -0.07 (-0.18, 0.04) | 0.72 |
| No | 12 | -0.49 (-0.87, -0.11) |  | 5 | -0.05 (-0.07, -0.02) |  |
| **Inclusion of the SP component** |  |  |  |  |  |  |
| Yes | 16 | -0.30 (-0.58, -0.02) | 0.44 | 9 | -0.04 (-0.08, -0.001) | 0.50 |
| No | 6 | -0.17 (-0.33, -0.01) |  | 2 | -0.08 (-0.20, 0.03) |  |
| Abbreviations: CI: confidence interval; SP: school policy; PA: physical activity; HE: health education | | | | | | |

| **Table S8 The list of excluded studies** | | |
| --- | --- | --- |
| **Author** | **Year** | **Reason for exclusion** |
| Haghani, etc | 2017 | Weight and height were reported by questionnaire, not measured. |
| Guo,etc | 2015 | With no comparison between intervention and control group |
| Boutelle, etc | 2017 | A family-based intervention |
| Annesi, etc | 2017 | This is not a school-based intervention. |
| Larsen, etc | 2016 | Not a school-based intervention but a day-camp project |
| Herget, etc | 2016 | Not a school-based intervention |
| Grillich, etc | 2016 | The intervention is aimed at teachers. |
| Spears-Lanoix, etc | 2015 | There is no control group |
| Daly, etc | 2016 | Participants are high school students. |
| Tarabashkina, etc | 2015 | Not a school-based intervention |
| Sutherland, etc | 2016 | Focus on cost-effectiveness of the intervention |
| Sanchez-Delgado, etc | 2015 | aged 18-25 |
| Bogart LM. etc | 2016 | 5 weeks' intervention, assessed at 2 years |
| Bhave S. etc | 2016 | Non-randomized non-blinded school-based intervention study |
| Beets MW. etc | 2016 | Not aimed at prevent overweight; only based on physical activity |
| Apolzan JW. etc | 2016 | Based on premenopausal women |
| Andrade S. etc | 2016 | Not aimed at prevent overweight; not including obesity-related outcomes |
| Anderson EL. etc | 2016 | Not aimed at prevent overweight; not including obesity-related outcomes |
| Ulrike M Müller. etc | 2016 | Not aimed at prevent overweight |
| Loth KA.etc | 2016 | Based on family intervention |
| B Dieris. etc | 2016 | Treatment |
| Roth K. etc | 2015 | Not aimed at prevent overweight; not including obesity-related outcomes |
| Greve, etc | 2015 | This is not an intervention focusing on nutrition and physical activity |
| NA | NA | No obesity-related outcomes |
| Eather, etc | 2016 | Less than 3 months (2 months) intervention |
| NA | NA | Community-based intervention |
| Christie, etc | 2015 | Community-based intervention |
| NA | NA | Only 8 weeks intervention |
| Bjelland, etc | 2011 | No obesity-related outcomes |
| Belcher, etc | 2015 | No obesity-related outcomes |
| Batalau, etc | 2017 | There is no control group and the analysis is irrelevant. |
| NA | NA | Community-based intervention |
| Larsen, etc | 2014 | Not a school-based intervention but a day-camp project in a remote camp location |
| Kobel, etc | 2014 | No obesity-related outcomes |
| Harder-Lauridsen, etc | 2014 | This is not a school-based intervention. |
| Damaso, etc | 2014 | Not a school-based intervention |
| Collins, etc | 2013 | No obesity-related outcomes |
| Cohen, etc | 2014 | No obesity-related outcomes |
| Cao, etc | 2014 | Not English Paper |
| Filho, etc | 2015 | No obesity-related outcomes |
| Anderson, etc | 2015 | Not a school-based intervention |
| Alberga, etc | 2015 | No obesity-related outcomes |
| Abraham, etc | 2015 | Not a school-based intervention |
| Miguel-Etayo, etc | 2015 | A family-based intervention |
| Sigal, etc | 2014 | Community-based intervention |
| Boodai, etc | 2014 | Not a school-based intervention |
| Bohman, etc | 2014 | Not relevant |
| Bergh, etc | 2014 | No obesity-related outcomes |
| Ackel-D'Elia, etc | 2013 | With no control group |
| Abraham, etc | 2015 | This is not a school-based intervention. |
| Roberts, etc | 2013 | 2 weeks intervention |
| Ha, etc | 2014 | No obesity-related outcomes |
| Kong, As.etc | 2013 | Not a randomized controlled trial |
| Jansson SP.etc | 2013 | Participants are not school student |
| Daniels SR. etc | 2005 | Not school-based |
| T Burrows. Etc | 2008 | Not school-based |
| Woo KS.etc | 2004 | Not school-based |
| Ribeiro MM, etc | 2005 | Not aimed at prevent overweight |
| Kelishadi R.etc | 2008 | Not aimed at prevent overweight |
| Leach RA,etc | 2008 | Not aimed at prevent overweight |
| Elloumi M.etc | 2009 | Not school-based |
| Shalitin S,etc | 2009 | Less than 3 month |
| Okely AD, etc | 2010 | Not school-based |
| Rocchini AP,etc | 1986 | Not aimed at prevent overweight |
| Schwingshandl J,etc | 1999 | Not school-based |
| Sung RYT, etc | 2002 | Less than 3 months |
| Janssen I,etc | 2010 | Not aimed at prevent overweight |
| Daniels SR, P.etc | 2011 | Not aimed at prevent overweight |
| Gutin B.etc | 2011 | Not a research |
| Elleuch, H. etc | 2013 | Not aimed at prevent overweight |
| Trinh, A. etc | 2013 | Not school-based |
| Buchan, Ds. etc | 2013 | Focus on cardiovascular disease risk |
| Brennan, L.etc | 2013 | Not school-based |
| Bianchini JAA. etc | 2013 | Not school-based |
| Alberga, As.etc | 2013 | Not aimed at prevent overweight |
| Adamo, Kb.etc | 2013 | Not aimed at prevent overweight |
| Magnusson KT. etc | 2012 | Not aimed at prevent overweight |
| Herbert, Pc. Etc | 2013 | Not including obesity-related outcomes |
| Daniels SR,etc | 2005 | Not a research |
| Steinberger J, etc | 2009 | Not a research; not aim at prevent overweight |
| McGovern L,etc | 2008 | Not aimed at prevent overweight |
| Oude Luttikhuis H, etc | 2009 | Not school-based |
| Weiss R,etc | 2004 | Not aimed at prevent overweight |
| Ben Ounis O,etc | 2010 | Not aimed at prevent overweight |
| Janicke DM,etc | 2008 | Not school-based |
| Kitzman-Ulrich H,etc | 2009 | Not school-based |
| McCallum Z, etc | 2007 | Not school-based |
| Park TG,etc | 2007 | Not aimed at prevent overweight |
| Rooney BL,etc | 2005 | Not aimed at prevent overweight |
| Shelton D,etc | 2007 | Not aimed at pupil |
| Díaz RG,etc | 2010 | Not school-based |
| Kalarchian MA,etc | 2009 | Not school-based |
| Kalavainen MP,etc | 2007 | Not school-based |
| Sabet Sarvestani R, etc | 2009 | Not school-based |
| Fullerton G,etc | 2007 | Not school based; not cluster RCTs |
| Balagopal P,etc | 2005 | Not school-based |
| Balagopal P,etc | 2005 | Not including obesity-related outcomes |
| Janicke DM, etc | 2009 | Not school-based |
| Stewart L, etc | 2005 | Not aimed at prevent overweight |
| Wake M,etc | 2008 | Not school-based |
| Kalavainen M, etc | 2009 | Not school-based |
| Shaw M,etc | 2009 | Not aimed at prevent overweight |
| Saelens BE,etc | 2002 | Not school-based |
| Braet C,etc | 1997 | Not school-based |
| Bell LM,etc | 2007 | Less than 3 month; not aimed at prevent overweight |
| Golan M,etc | 2006 | Not school-based |
| DeBar LL. etc | 2012 | Not school-based |
| Crespo NC.etc | 2012 | Not school-based |
| Coleman, etc | 2012 | The intervention is not aimed at students. |
| NA | NA | An eight-week intervention |
| Janssen, etc | 2012 | No obesity-related outcomes |
| Heer, etc | 2011 | With no comparison between intervention and control groups |
| Glazebrook, etc | 2011 | An 10-week intervention |
| Doyle-Baker, etc | 2011 | An 10-week intervention |
| Toulabi, etc | 2010 | A high school-based intervention |
| Chen, etc | 2011 | A family-based intervention |
| Bundy, etc | 2017 | No obesity-related outcomes |
| Buchan, etc | 2011 | Not a school-based intervention and only lasts 7 weeks |
| Bryant, etc | 2011 | Delivered in a community setting |
| NA | NA | An 8-week intervention |
| Berkowitz, etc | 2011 | A family-based lifestyle modification program |
| Backlund, etc | 2011 | A family-based intervention |
| Backlund, etc | 2011 | A family-based intervention |
| Heijden, etc | 2010 | No obesity-related outcomes |
| Hartmann, etc | 2010 | No obesity-related outcomes |
| Francis, etc | 2010 | No obesity-related outcomes |
| Dzewaltowski, etc | 2010 | This is a cross-sectional trial |
| Diaz, etc | 2010 | A family-centered program |
| Christiansen, etc | 2010 | Not a school-based intervention and age 18-45. |
| NA | NA | This is a conference abstract. |
| Byrd-Williams, etc | 2010 | Not a school-based intervention |
| Brownlee, etc | 2010 | Aged 18-65 |
| Bravender, etc | 2010 | A 2-month intervention |
| Sun, etc | 2011 | A 10-week intervention |
| Plachta-Danielzik, etc | 2011 | This is not an RTC, but a cross-sectional study, measuring different samples every year. |
| NA | NA | Not a school-based intervention |
| Kain, etc | 2008 | This is a non-randomized controlled trial |
| Burrows, etc | 2008 | No obesity-related outcomes |
| Benson AC. etc | 2008 | Intervention less than 3 month |
| Alves, Jg. etc | 2008 | Not school-based |
| Abood, Da. etc | 2008 | Not including obesity-related outcomes |
| Tak YR. etc | 2007 | Intervention less than 3 month |
| Boddy, Lm. etc | 2012 | Not including obesity-related outcomes |
| Bergh IH.etc | 2012 | Not including obesity-related outcomes |
| Antoine B.etc | 2012 | Not school-based; not aimed at prevent overweight |
| Barbeau P.etc | 2007 | Not school-based |
| Austin SB.etc | 2007 | Not including obesity-related outcomes |
| Warren JM.etc | 2003 | Not including obesity-related outcomes |
| Duncan MJ.etc | 2009 | A 6-week intervention |
| Christiansen, T.etc | 2009 | Not school-based |
| Carrel, Al.etc | 2009 | Not including obesity-related outcomes |
| Ben, Ounis O. etc | 2009 | A 2-month intervention |
| Bayer, O.etc | 2009 | Not including obesity-related outcomes |
| Kain, J. etc | 2008 | Non-randomized |
| Baranowski, T. etc | 2003 | Not school-based |
| Eliakim, A. etc | 2002 | Non-randomized |
| Braet, C. etc | 1997 | Treatment |
| Harrell, Js. etc | 1996 | Not school-based |
| Donnelly, Je. etc | 1996 | Not including obesity-related outcomes |
| Burke V. etc | 1996 | Not including obesity-related outcomes |
| Jurg, Me. etc | 2006 | Not including obesity-related outcomes |
| Harrison, M. etc | 2006 | Not including obesity-related outcomes not aimed at prevent overweight |
| Haines, etc | 2006 | No obesity-related outcomes |
| Ebbeling, etc | 2006 | A home-centered intervention |
| Horn, etc | 2005 | No obesity-related outcomes |
| Nemet D, etc | 2005 | An intervention set on outpatient |
| Ames, etc | 2005 | Aged 18-30 |
| Simon, etc | 2004 | No obesity-related outcomes |
| Graf, etc | 2006 | This is an outpatient intervention. |
| Duncan, etc | 2009 | A 6-week intervention |
| Mihas, etc | 2009 | This is aimed at high school students. |
| Simon, etc | 2008 | Without BMI or BMI-Z outcomes |
| Bayne-Smith, etc | 2004 | This is aimed at high school students. |
| Dennison, etc | 2004 | This is aimed at preschool students. |
| Fitzgibbon, etc | 2006 | This is aimed at preschool students. |
| Flores, etc | 1995 | Without BMI or BMI-Z outcomes |
| Harrell, etc | 1996 | This is an 8-week intervention |
| Johnston, etc | 2010 | No comparison |
| Matvienko, etc | 2010 | A 4-week intervention |
| Melnyk, etc | 2007 | High school students; and only a 9-week intervention |
| Nader, etc | 1999 | Not an intervention |
| Stock, etc | 2007 | This is a non-randomized controlled trial |
| Mo-suwan, etc | 1998 | Aimed at kindergarten children |
| Wong, etc | 2008 | This is a treatment intervention. |
| Neumark-Sztainer, etc | 2003 | The intervention is aimed at high school students. |
| Story, etc | 2003 | This is not a school-based intervention but a community-based intervention. |
| Eliakim, etc | 2007 | This is aimed at preschool students. |
| Johnston, etc | 2007 | There is no control group, SH group also received intervention. |
| Johnston, etc | 2010 | No comparison |
| Gentile, etc | 2009 | This is not a school-based intervention |
| Johnston, etc | 2010 | No comparison |
| Almas, etc | 2013 | This is a non-randomized controlled trial |
| Croker, etc | 2012 | This is a family-based intervention |
| Dzewaltowski, etc | 2010 | This is a cross-sectional trial, not randomized, this is the same article as #150 |
| Estabrooks, etc | 2009 | This is a family-based intervention |
| Gentile, etc | 2009 | Duplicate |
| Golley, etc | 2007 | This is a family-focused intervention. |
| Graf, etc | 2005 | This is a non-randomized controlled trial |
| Kalarchian, etc | 2009 | This is a family-based intervention. |
| Kalavainen, etc | 2007 | This is a family-based intervention. |
| Sacher, etc | 2010 | This is a family-based intervention. |
| Wafa, etc | 2011 | This is not a school-based intervention. |
| Waling, etc | 2010 | This is not a school-based intervention. |
| Elder, etc | 2011 | No obesity-related outcomes |
| Dreyhaupt, etc | 2012 | A protocol and study design( after further searching the outcome is recorded, but without obesity-related outcomes.) |
| Baranowski, etc | 2012 | An introduction of systematic review and discussion about its meaning. |
| Kibbe, etc | 2011 | A summary of different studies using the same intervention project. |
| Shalitin, etc | 2009 | No control group |
| Skybo, etc | 2002 | This is not a randomized study |
| Grey, etc | 2004 | This is a family-centered intervention. |
| Warren, etc | 2003 | There is no comparison. |
| Kain, etc | 2004 | The method is not randomized. |
| Sallis, etc | 1993 | This is not fully randomized. |
| Sahota, etc | 2001 | The analysis is not relevant, no enough data. |
| Sallis, etc | 2003 | Height and weight are self-reported by students. |
| Gortmaker, etc | 1999 | No obesity-related outcomes |
| Mo-suwan, etc | 1998 | Aimed at kindergarten children |
| Burke, etc | 1998 | Without BMI or BMI-Z outcomes |
| Robinson, etc | 2003 | Not a school-based intervention (but in a community center) |
| Harrell, etc | 2005 | Not randomized |
| Woo, etc | 2004 | This is a 6-week intervention |
| Shaibi, etc | 2006 | This is not a school-based intervention. (but in a clinic) |
| Alves, etc | 2008 | Not a school-based intervention |
| Draper, etc | 2010 | This is a paper describing the development of a specific intervention project. |
| Duffy, etc | 1993 | This is not a school-based intervention. |
| Epstein, etc | 2001 | This is a family-based intervention. |
| Epstein, etc | 2004 | This is a family-based intervention. |
| Goldfield, etc | 2001 | This is a family-based intervention and the article focuses on the cost-effectiveness. |
| Hills, etc | 1988 | This is not a school-based intervention. (but in a clinic) |
| Lansky, etc | 1982 | There is no control group |
| Mellin, etc | 1987 | This is not a school-based intervention. (but in a clinic) |
| Saelens, etc | 2002 | This is not a school-based intervention. (but in a clinic) |
| Sondike, etc | 2003 | This is not a school-based intervention. |
| Nova, etc | 2001 | This is not a school-based intervention and the intervention lasts for only 6 weeks. |
| Golan, etc | 1998 | This is a family-based intervention. |
| Gutin, etc | 2002 | This is not a school-based intervention. |
| Senediak, etc | 1985 | This is a family-based intervention. |
| Rocchini, etc | 1987 | This is not a school-based intervention. |
| Epstein, etc | 2000 | This is a family-based intervention. |
| Braet, etc | 2000 | This is not school-based intervention.(outpatient/summer camp settings) |
| Epstein, etc | 1994 | This is a family-based intervention. |
| Golan, etc | 2004 | This is a family-based intervention. |
| Ludwig, etc | 2001 | There is no intervention. (not relevant) |
| Amaro, etc | 2006 | This is not nutrition and/or physical activity intervention. |
| Baranowski, etc | 2003 | This is not a school-based intervention (but set in a summer camp and at home). |
| Beech, etc | 2003 | This is not school-based intervention (but is set in a community center). |
| Coleman, etc | 2005 | This is not a fully randomized intervention. |
| Dennison, etc | 2004 | This is aimed at preschool students; duplicate |
| Ebbeling, etc | 2006 | A home-centered intervention; duplicate |
| Epstein, etc | 2001 | This is a family-based intervention. |
| Fernandes, etc | 2009 | There is no English version article. (is not a school-based intervention according to the abstract) |
| Fitzgibbon, etc | 2002 | This is aimed at preschool students. |
| Fitzgibbon, etc | 2006 | This is aimed at preschool students. |
| Haerens, etc | 2006 | No obesity-related outcomes |
| Harvey-Berino, etc | 2003 | This is aimed at preschool students. |
| Jouret, etc | 2009 | The intervention is aimed at kindergarten students. |
| Marcias-Cervantes, etc | 2009 | This is not a school-based intervention. (but in a local sports center) |
| Mo-suwan, etc | 1998 | aimed at kindergarten children (the same article as #224_38) |
| Muller, etc | 2001 | There is no control group |
| Paineau, etc | 2008 | This is a family-based intervention. |
| Pangrazi, etc | 2003 | This is not a fully randomized intervention. |
| Pate, etc | 2005 | This is aimed at high school students. |
| Patrick, etc | 2006 | This is a home-based intervention. |
| Reilly, etc | 2006 | This is aimed at preschool students. |
| Robbins, etc | 2006 | No obesity-related outcomes |
| Robinson, etc | 2003 | Not a school-based intervention (but in a community center); duplicate |
| Rodearmel, etc | 2006 | This is a family-based intervention. |
| Salmon, etc | 2005 | No outcome data (not published) |
| Sanigorski, etc | 2008 | Age is not qualified. (preschool students) |
| Spiegel, etc | 2006 | Without BMI or BMI-Z outcomes |
| Stolley, etc | 1997 | This is not a school-based intervention. |
| Rochon, etc | 2003 | This is not a school-based intervention. |
| Taylor, etc | 2008 | This is a community-based intervention but not a school-based intervention. |
| Taylor, etc | 2007 | This is a community-based intervention but not a school-based intervention. |
| Webber, etc | 2008 | Not relevant intervention |
| Stolley, etc | 1997 | This is not a school-based intervention. |
| Mo-suwan, etc | 1998 | Aimed at kindergarten children |
| Donnelly, etc | 1996 | Not randomized |
| Gutin, etc | 1997 | This is not school-based intervention. |
| Gutin, etc | 2002 | This is not a school-based intervention. |
| Ferguson, etc | 1999 | This is not a school-based intervention. |
| Woo, etc | 2004 | this is not a school-based intervention.(carried out in a hospital) |
| Emes, etc | 1990 | This is not a school-based intervention. |
| Faith, etc | 2001 | This is a family-based intervention. |
| Katch, etc | 1988 | This is not a school-based intervention. |
| Stice E,etc | 2006 | Intervention not aimed at pupil |
| Lin JS,etc | 2014 | Not aimed at preventing overweight |
| Martin Cantera C,etc | 2015 | Not aimed at preventing overweight |
| Secades-Villa R,etc | 2009 | Not aimed at preventing overweight |
| Lyles CM,etc | 2007 | Not aimed at preventing overweight |
| Glasgow RE,etc | 1999 | just a checklist |
| Ramezani, A. etc | 2017 | Not aimed at preventing overweight |
| Sorof J,etc | 2002 | Not aimed at preventing overweight |
| Rosner B,etc | 2013 | Not aimed at preventing overweight |
| Bayer O,etc | 2010 | Not aimed at preventing overweight |
| Falkner B,etc | 2011 | Not aimed at preventing overweight |
| Ferreira I,etc | 2012 | Not aimed at preventing overweight |
| Li S,etc | 2004 | Not aimed at preventing overweight |
| Li X,etc | 2004 | Not aimed at preventing overweight |
| Must A, etc | 1992 | Not aimed at preventing overweight |
| Must A,etc | 2012 | Not aimed at preventing overweight |
| Franks PW,etc | 2010 | Not aimed at preventing overweight |
| Lawlor DA,etc | 2006 | Not aimed at preventing overweight |
| Kelder SH,etc | 1994 | Not aimed at preventing overweight |
| Hayman LL,etc | 2004 | Not aimed at preventing overweight |
| Waters E,etc | 2011 | Not a research |
| Cai L,etc | 2014 | Not including obesity-related outcomes |
| Van den Noortgate W,etc | 2013 | Not aimed at preventing overweight |
| Chan EK,etc | 2012 | Not aimed at preventing overweight |
| Crespo NC,etc | 2012 | Not school-based |
|  |  | The study focused on preschool students |
| Eather N,etc | 2013 | Not aimed at preventing overweight |
| Gentile DA,etc | 2009 | Not school-based |
| Hansen HS,etc | 1991 | Not aimed at preventing overweight |
| Harrell JS,etc | 1999 | Not aimed at preventing overweight |
| Harrison M,etc | 2006 | Not randomized |
| Hoffman JA,etc | 2011 | Not including obesity-related outcomes |
| Jouret B,etc | 2009 | Aimed at preschool children |
| Manios Y,etc | 1999 | BMI outcomes reported by questionnaire, not measured |
| Manios Y,etc | 2002 | Not aimed at prevent overweight |
| Muckelbauer R,etc | 2009 | not including obesity-related outcomes |
| Niederer I,etc | 2013 | Aimed at preschool children |
| Plachta-Danielzik S,etc | 2011 | An follow-up study |
| Rausch Herscovici C,etc | 2013 | Not including obesity-related outcomes |
| Reed KE,etc | 2008 | Not aimed at prevent overweight |
| Reilly JJ,etc | 2006 | Aimed at preschool children |
| Rosario R,etc | 2013 | Not aimed at prevent overweight |
| Sacchetti R,etc | 2013 | Reported by questionnaire, not measured |
| NA |  | Without BMI or BMI-Z outcomes |
| NA |  | This study focused on kindergarten children. |
| Toruner EK,etc | 2010 | Less than 3 month |
| Vandongen R,etc | 1995 | Not aimed at preventing overweight |
| Walther C,etc | 2009 | Not aimed at preventing overweight |
| Yin Z,etc | 2005 | Not including obesity-related outcomes |
| MacKelvie, etc | 2003 | The object is not treating or preventing weight gaining. |
| Ahamed, etc | 2007 | Not relevant |
| NA | NA | Without BMI or BMI-Z outcomes |
| Gong, etc | 2014 | The aim of the intervention is to elevate iron but not to lose weight. |
| Li, etc | 2014 | This is a non-randomized controlled trial |
| Meng, etc | 2013 | This focuses on cost-effectiveness of the intervention. |
| Haerens, etc | 1991 | This is not a school-based intervention. |
| Luepker, etc |  | There is no comparison. |
| Manios, etc | 1998 | No obesity-related outcomes |
| Ritenbaugh, etc | 2003 | This is aimed at high school students. |
| Sallis, etc | 1997 | This is not a fully randomized intervention. |
| Trevino, etc | 2005 | No obesity-related outcomes comparison |
| Wardle, etc | 2007 | This is a non-randomized controlled trial |
| Bergh, etc | 2012 | No obesity-related outcomes |
| Gorely, etc | 2009 | This is a non-randomized controlled trial |
| Shrewsbury, etc | 2011 | This is a community-based intervention but not a school-based intervention. |
| Nguyen, etc | 2013 | This is a community-based intervention but not a school-based intervention. |
| Bauer, etc | 2010 | This is not a school-based intervention. |
| Shrewsbury, etc | 2009 | This is a community-based intervention but not a school-based intervention. |
| Chehab, etc | 2007 | This intervention is aimed at high school students. |
| Devault, etc | 2009 | No obesity-related outcomes |
| Hollar, etc | 2010 | This is a non-randomized controlled trial |
| Johnston, etc | 2012 | There is no control group |
| Manger, etc | 2012 | This is not a randomized study, and school chooses to be intervention school voluntarily. |
| Melnyk, etc | 2009 | No valid and obesity-related outcomes |
| Pbert, etc | 2013 | The intervention is aimed at high school students. |
| Walther, etc | 2009 | This intervention is not aimed to lose weight. |
| Wong, etc | 2013 | This is not an intervention using PA or nutrition methods. |
| Doyle, etc | 2008 | This is not a school-based intervention. |
| Jones, etc | 2008 | This is aimed at high school students. |
| Mauriello, etc | 2010 | This is aimed at high school students. |
| Maes, etc | 2011 | No obesity-related outcomes |
| Casazza, etc | 2007 | The intervention lasts 11 weeks. |
| Whittemore, etc |  | This is aimed at high school students. |
| Williamson, etc | 2006 | This is not a school-based intervention. |
| Frenn, etc | 2003 | No obesity-related outcomes |
| Frenn, etc | 2005 | No obesity-related outcomes |
| Noia, etc | 2008 | No-obesity related outcomes |
| Mangunkusumo, etc | 2006 | No obesity-related outcomes |
| Haerens, etc | 2007 | No obesity-related outcomes |
| Ezendam, etc | 2012 | The intervention lasts for 10 weeks |
| Yu, etc | 2005 | This is not a school-based intervention but implemented during a summer vacation to a small portion of students. |
| Burrows, etc | 2010 | This is not a school-based intervention but focuses on family. |
| Ribeiro, etc | 2005 | This is not a school-based intervention. |
| Kelishadi, etc | 2008 | This is not a school-based intervention and there is no control group. |
| Leach, etc | 2008 | This is not a school-based intervention. |
| Davis, etc | 2009 | This is not a school-based intervention. |
| Elloumi, etc | 2009 | This intervention lasts for only 2 months. |
| Okely, etc | 2010 | This is not a school-based intervention. |
| Epstein, etc | 1990 | This is a family-based intervention |
| Schwingshandl, etc | 1999 | This is not a school-based intervention but took place in a public gym. |
| Verstraete, etc | 2007 | No obesity-related outcomes |
| Young, etc | 2006 | The intervention is aimed at high school students. |
| Henaghan, etc | 2008 | The intervention lasts for only 9 weeks. |
| McManus, etc | 2008 | The intervention lasts for less than 3 weeks. |
| Elberg, etc | 2004 | Not relevant |
| Suskind, etc | 2000 | This article contains two intervention projects, and both of them are not school-based. |
| Sudi, etc | 2001 | Not relevant |
| Sung, etc | 2002 | The program lasts for only 6 weeks. |
| Tsiros, etc | 2008 | This is not a school-based intervention. |
| Tjonna, etc | 2009 | This is not a school-based intervention. (but in a clinic) |
| Magarey, etc | 2011 | This is a family focused intervention. |
| Savoye, etc | 2011 | This is a family focused intervention. |
| Risica PM | 2019 | only protocol, not providing main results |
| Sánchez-López M | 2019 | only protocol, not providing main results |
| Aittasalo M | 2019 | Studies not assessing BMI indices |
| Dong L | 2019 | Irrelevant |
| Karmali S | 2019 | only protocol, not providing main results |
| Toussaint N | 2019 | only protocol, not providing main results |
| Tucker JM | 2019 | Age range not in Intervention lasting for shorter than 3 months~18 years |
| Luque V | 2019 | Not individual or cluster-RCT0 |
| Nguo K | 2019 | No control |
| Milliken S | 2019 | Irrelevant |
| Hamulka J | 2018 | Others |
| Wadolowska L | 2019 | Not individual or cluster-RCT |
| Byrne ME | 2019 | Irrelevant |
| Ports KA | 2019 | Irrelevant |
| Liao XP | 2019 | Irrelevant |
| Mastrandrea LD | 2019 | Irrelevant |
| Wright CM | 2019 | Irrelevant |
| Kunath J | 2019 | Irrelevant |
| Seo YG | 2019 | Intervention focused only on overweight or obese students |
| Volger S | 2018 | Irrelevant |
| López-Alarcón M | 2019 | Irrelevant |
| Schmidt R | 2018 | Irrelevant |
| Connolly SD | 2018 | Irrelevant |
| Bean MK | 2019 | Irrelevant |
| Yang YJ | 2019 | Irrelevant |
| Nehus E | 2019 | Irrelevant |
| Er V | 2018 | Age range not in Intervention lasting for shorter than 3 months~18 years |
| Bozzi Cionci N | 2018 | Irrelevant |
| Di Genova L | 2018 | Irrelevant |
| Hayes JF | 2018 | Irrelevant |
| Trude ACB | 2018 | Studies not assessing BMI indices |
| Roberge JB | 2018 | Irrelevant |
| Thivel D | 2019 | Irrelevant |
| Murray M | 2018 | Intervention focused only on overweight or obese students |
| Eidelman AI | 2018 | Irrelevant |
| Ojeda-Rodríguez A | 2018 | Intervention focused only on overweight or obese students |
| Weihrauch-Blüher S | 2018 | Irrelevant |
| Michaliszyn SF | 2018 | Intervention focused only on overweight or obese students |
| Kumar S | 2018 | Irrelevant |
| Lee EY | 2018 | Irrelevant |
| Shakhnovich V | 2019 | Intervention focused only on overweight or obese students |
| Crespo NC | 2018 | Intervention focused only on overweight or obese students |
| Jastreboff AM | 2018 | Irrelevant |
| Rodriguez-Ventura A | 2018 | Intervention focused only on overweight or obese students |
| Kamel M | 2018 | Irrelevant |
| Julian V | 2018 | Irrelevant |
| Ekambareshwar M | 2018 | Irrelevant |
| Santos DA | 2019 | Irrelevant |
| Claesson IM | 2018 | Irrelevant |
| Naets T | 2018 | only protocol, not providing main results |
| Cloutier MM | 2018 | Intervention focused only on overweight or obese students |
| Damanhoury S | 2018 | Irrelevant |
| Chan DF | 2018 | Intervention focused only on overweight or obese students |
| Urbina EM | 2018 | Irrelevant |
| Vidmar AP | 2019 | Intervention focused only on overweight or obese students |
| Emmanouil CC | 2018 | Intervention focused only on overweight or obese students |
| Paul IM | 2018 | Age range not in Intervention lasting for shorter than 3 months~18 years |
| Barkin SL | 2018 | Age range not in Intervention lasting for shorter than 3 months~18 years |
| Grandone A | 2018 | Irrelevant |
| Villarosa AR | 2018 | Irrelevant |
| Pratt JSA | 2018 | Irrelevant |
| Ahmad N | 2018 | Intervention focused only on overweight or obese students |
| Hayes JF | 2018 | Intervention focused only on overweight or obese students |
| Valerio G | 2018 | Irrelevant |
| Rocha EPAA | 2018 | Irrelevant |
| Staiano AE | 2018 | Intervention focused only on overweight or obese students |
| Gómez SF | 2018 | Irrelevant |
| Specht IO | 2018 | Irrelevant |
| Savage JS | 2018 | Irrelevant |
| Albert Pérez E | 2018 | Irrelevant |
| Haines J | 2018 | Age range not in Intervention lasting for shorter than 3 months~18 years |
| Naude CE | 2018 | Irrelevant |
| Wang LX | 2018 | Irrelevant |
| Wachira LM | 2018 | Irrelevant |
| Khan AS | 2018 | Irrelevant |
| Bean MK | 2018 | Irrelevant |
| Ashley P | 2018 | Irrelevant |
| DE Groote E | 2018 | Intervention focused only on overweight or obese students |
| Rauber SB | 2018 | Intervention focused only on overweight or obese students |
| Warschburger P | 2018 | Intervention focused only on overweight or obese students |
| Karasz A | 2018 | Irrelevant |
| Sen M | 2018 | Intervention focused only on overweight or obese students |
| Flores-Peña Y | 2018 | only protocol, not providing main results |
| Lanigan J | 2018 | Irrelevant |
| Delisle Nyström C | 2018 | Age range not in Intervention lasting for shorter than 3 months~18 years |
| Trude ACB | 2018 | Irrelevant |
| Bagherniya M | 2018 | Intervention focused only on overweight or obese students |
| Quesada D | 2018 | Irrelevant |
| Anderson YC | 2018 | Irrelevant |
| Dayton K | 2018 | Irrelevant |
| Smith LH | 2018 | only protocol, not providing main results |
| Karacabeyli D | 2018 | Irrelevant |
| Freira S | 2018 | Intervention focused only on overweight or obese students |
| McPherson AC | 2018 | Irrelevant |
| Nissensohn M | 2018 | Irrelevant |
| Bruñó A | 2018 | Intervention focused only on overweight or obese students |
| Cominato L | 2018 | Irrelevant |
| Eidlitz Markus T | 2018 | Irrelevant |
| Kral TVE | 2018 | Irrelevant |
| Hemmingsson E | 2018 | Irrelevant |
| Zolotarjova J | 2018 | Intervention focused only on overweight or obese students |
| Omran J | 2018 | Irrelevant |
| Lerman J | 2018 | Irrelevant |
| Delgado-Floody P | 2018 | Intervention focused only on overweight or obese students |
| Kobes A | 2018 | Irrelevant |
| Butler ÉM | 2018 | Irrelevant |
| Sharma AM | 2018 | Irrelevant |
| McMorrow AM | 2018 | Intervention focused only on overweight or obese students |
| van der Valk ES | 2018 | Irrelevant |
| Bocca G | 2018 | Irrelevant |
| Tanskey LA | 2018 | Irrelevant |
| Brar PC | 2018 | Irrelevant |
| Stanford FC | 2018 | Irrelevant |
| Elinder LS | 2018 | only protocol, not providing main results |
| Belfort-DeAguiar R | 2018 | Irrelevant |
| Byrd AS | 2018 | Irrelevant |
| Kawabata M | 2018 | only protocol, not providing main results |
| Ersfjord EMI | 2018 | Irrelevant |
| Levran N | 2018 | Irrelevant |
| Smith JD | 2018 | Irrelevant |
| Nardo Junior N | 2018 | Irrelevant |
| Traub M | 2018 | Irrelevant |
| Adams EL | 2018 | Irrelevant |
| Croyden DL | 2018 | Irrelevant |
| Norman Å | 2018 | Irrelevant |
| Elmesmari R | 2018 | Irrelevant |
| Snowden J | 2018 | Irrelevant |
| Martin A | 2018 | Irrelevant |
| Jones RB | 2018 | Irrelevant |
| Mustila T | 2018 | Irrelevant |
| Shih KC | 2018 | Irrelevant |
| Mann JP | 2018 | Irrelevant |
| Fisher A | 2018 | Irrelevant |
| Yang Q | 2018 | Intervention lasting for shorter than 3 months |
| Medrano M | 2018 | Irrelevant |
| Ingul CB | 2018 | Intervention focused only on overweight or obese students |
| Panca M | 2018 | Irrelevant |
| Whooten RC | 2018 | Not individual or cluster-RCT |
| Naude CE | 2018 | Irrelevant |
| Bussiek PV | 2018 | only protocol, not providing main results |
| Ruiz RM | 2018 | Irrelevant |
| Davidson K | 2018 | Irrelevant |
| Archibald AJ | 2018 | Irrelevant |
| Adab P | 2018 | Duplicates |
| Cairo SB | 2018 | Irrelevant |
| Riesche L | 2018 | Irrelevant |
| Chao AM | 2018 | Irrelevant |
| Cunha DB | 2017 | only protocol, not providing main results |
| Shakhnovich V | 2018 | Irrelevant |
| Mirotta JA | 2018 | Age range not in Intervention lasting for shorter than 3 months~18 years |
| Koo HC | 2018 | Not individual or cluster-RCT |
| Yan H | 2018 | Irrelevant |
| Chung ST | 2018 | Irrelevant |
| Martin A | 2018 | Intervention focused only on overweight or obese students |
| Bishop JC | 2018 | Irrelevant |
| Latorre-Román PA | 2018 | Age range not in Intervention lasting for shorter than 3 months~18 years |
| Wylie-Rosett J | 2018 | Age range not in Intervention lasting for shorter than 3 months~18 yearsAge range not in Intervention lasting for shorter than 3 months~18 years |
| Viggiano E | 2018 | Studies not providing means and variance |
| Stern M | 2018 | Age range not in Intervention lasting for shorter than 3 months~18 yearsAge range not in Intervention lasting for shorter than 3 months~18 years |
| Nehus E | 2018 | Age range not in Intervention lasting for shorter than 3 months~18 yearsAge range not in Intervention lasting for shorter than 3 months~18 years |
| Yackobovitch-Gavan M | 2018 | Age range not in Intervention lasting for shorter than 3 months~18 yearsAge range not in Intervention lasting for shorter than 3 months~18 years |
| Ordway MR | 2018 | Age range not in Intervention lasting for shorter than 3 months~18 years |
| Heo M | 2018 | Intervention focused only on overweight or obese students |
| Smith JD | 2018 | only protocol, not providing main results |
| Greydanus DE | 2018 | Irrelevant |
| Breheny K | 2018 | only protocol, not providing main results |
| Stookey JD | 2017 | Age range not in Intervention lasting for shorter than 3 months~18 years |
| Kahan S | 2018 | Irrelevant |
| Liu M | 2018 | Intervention focused only on overweight or obese students |
| Llauradó E | 2018 | Duplicates |
| Harrison SE | 2018 | Irrelevant |
| Perepezko K | 2018 | Irrelevant |
| Bagherniya M | 2018 | Irrelevant |
| Horodynski MA | 2018 | Irrelevant |
| Reifsnider E | 2017 | Age range not in Intervention lasting for shorter than 3 months~18 years |
| Kang DW | 2018 | Intervention focused only on overweight or obese students |
| Pedroso FE | 2018 | Irrelevant |
| Hoffman J | 2018 | Intervention focused only on overweight or obese students |
| Enö Persson J | 2018 | Age range not in Intervention lasting for shorter than 3 months~18 years |
| Matvienko-Sikar K | 2018 | Age range not in Intervention lasting for shorter than 3 months~18 years |
| Foster C | 2018 | Irrelevant |
| Foster BA | 2018 | Irrelevant |
| Lim J | 2018 | Irrelevant |
| Geary NA | 2017 | Irrelevant |
| de Lira CT | 2017 | Intervention focused only on overweight or obese students |
| Miguel-Berges ML | 2017 | Irrelevant |
| Murugesan S | 2018 | Irrelevant |
| Li B | 2017 | only protocol, not providing main results |
| Moores CJ | 2017 | Irrelevant |
| Wolfenden L | 2017 |  |
| Lloyd J | 2017 | Duplicates |
| Koves IH | 2017 | Irrelevant |
| Aggarwal B | 2018 | Irrelevant |
| Xiong Y | 2017 | Irrelevant |
| Khokhar A | 2017 | Irrelevant |
| Cheng L | 2017 | Irrelevant |
| Ward DS | 2017 | Irrelevant |
| Rastogi D | 2017 | Irrelevant |
| Anderson KL | 2018 | Irrelevant |
| Stark LJ | 2017 | Age range not in Intervention lasting for shorter than 3 months~18 years |
| Byrd-Bredbenner C | 2017 | Irrelevant |
| Heerman WJ | 2017 | Duplicates |
| Patel N | 2017 | Irrelevant |
| Baskaran C | 2018 | Irrelevant |
| Hu Y | 2017 | Age range not in Intervention lasting for shorter than 3 months~18 years |
| Wright JA | 2018 | Irrelevant |
| Chen FT | 2017 | Intervention focused only on overweight or obese students |
| Perry RA | 2017 | Intervention focused only on overweight or obese students |
| Wong SS | 2017 | Irrelevant |
| Chuensiri N | 2018 | Intervention focused only on overweight or obese students |
| Sharifi M | 2017 | Irrelevant |
| Wilfley DE | 2017 | Irrelevant |
| Qi L | 2017 | Intervention focused only on overweight or obese students |
| Poti JM | 2017 | Irrelevant |
| Sundström P | 2017 | Irrelevant |
| Schadler KL | 2017 | Irrelevant |
| Bleich SN | 2018 | Duplicates |
| Buttitta M | 2017 | Irrelevant |
| Anderson YC | 2017 | Duplicates |
| Hollister EB | 2017 | Age range not in Intervention lasting for shorter than 3 months~18 years |
| Wang KW | 2018 | Irrelevant |
| Welbourn R | 2018 | Irrelevant |
| Shafei AE | 2018 | Duplicates |
| Armstrong S | 2017 | Irrelevant |
| Amin S | 2018 | Irrelevant |
| Hohman EE | 2018 | Age range not in Intervention lasting for shorter than 3 months~18 years |
| Cui X | 2018 | Irrelevant |
| Knowlden AP | 2018 | Irrelevant |
| Lichtveld K | 2017 | Irrelevant |
| Dinkel D | 2018 | Irrelevant |
| An R | 2017 | Irrelevant |
| Kebbe M | 2017 | Irrelevant |
| Fidler Mis N | 2017 | Irrelevant |
| Graziano PA | 2017 | Not individual or cluster-RCT |
| Hens W | 2017 | Intervention focused only on overweight or obese students |
| Rohde JF | 2017 | Age range not in Intervention lasting for shorter than 3 months~18 years |
| Cohen TR | 2017 | Intervention focused only on overweight or obese students |
| Burchett HED | 2017 | Irrelevant |
| Dias KA | 2018 | Intervention focused only on overweight or obese students |
| Tyson N | 2018 | Irrelevant |
| Kaakinen P | 2017 | Intervention focused only on overweight or obese students |
| Banna J | 2017 | Irrelevant |
| Kahrass H | 2017 | Irrelevant |
| Dunker KLL | 2018 | Studies not providing means and variance |
| Tenório TRS | 2017 | Intervention focused only on overweight or obese students |
| Chaplais E | 2017 | Intervention focused only on overweight or obese students |
| Marti A | 2018 | Intervention focused only on overweight or obese students |
| Han A | 2018 | Intervention focused only on overweight or obese students |
| An R | 2017 | Irrelevant |
| Miguel-Berges ML | 2018 | Irrelevant |
| Costa CS | 2018 | Irrelevant |
| Berkowitz RI | 2017 | Not individual or cluster-RCT0 |
| Sauder KA | 2018 | Not individual or cluster-RCT0 |
| Shoar S | 2018 | Irrelevant |
| Kim Y | 2017 | Irrelevant |
| Hayes JF | 2017 | Irrelevant |
| Kominiarek MA | 2017 | Irrelevant |
| Relvas M | 2017 | Irrelevant |
| Nafiu OO | 2018 | Irrelevant |
| Menezo Y | 2017 | Irrelevant |
| Potter C | 2018 | Irrelevant |
| Symon B | 2017 | Irrelevant |
| Kim J | 2017 | Irrelevant |
| Al Khalifah RA | 2017 | Irrelevant |
| Agostoni C | 2017 | Irrelevant |
| García-Hermoso A | 2018 | Irrelevant |
| Mameli C | 2018 | Irrelevant |
| Hull PC | 2018 | Irrelevant |
| Dawson AM | 2018 | Irrelevant |
| Byrne JLS | 2018 | Irrelevant |
| Arora T | 2017 | Irrelevant |
| Hutchins J | 2017 | Irrelevant |
| Galescu OA | 2018 | Irrelevant |
| Natale RA | 2017 | Irrelevant |
| Rhodes ET | 2017 | Irrelevant |
| Taylor JH | 2017 | Intervention focused only on overweight or obese students |
| Ochoa-Avilés A | 2017 | Studies not assessing BMI indices |
| Sutherland RL | 2017 | Studies not assessing BMI indices |
| Ickovics JR | 2019 | Studies not assessing BMI indices |


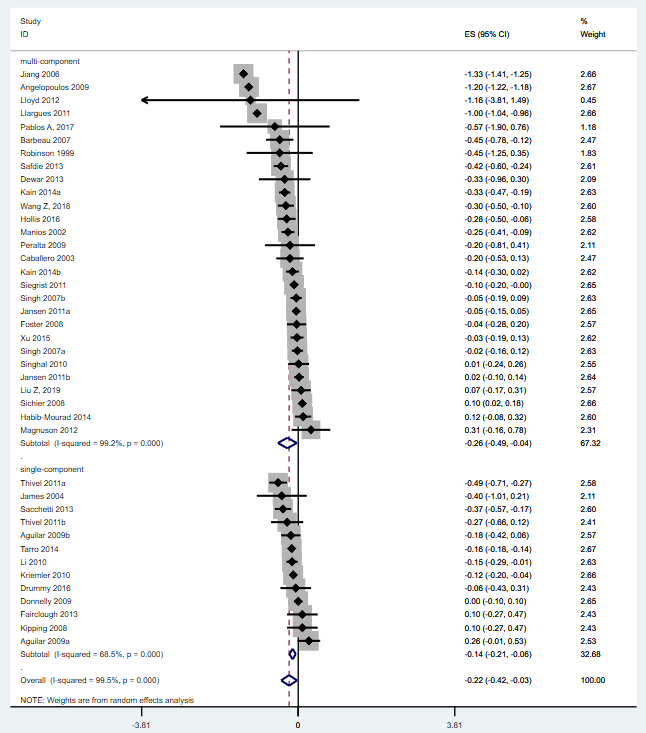


**Figure S1 Pooled intervention effect after excluding heterogeneous studies (BMI)**


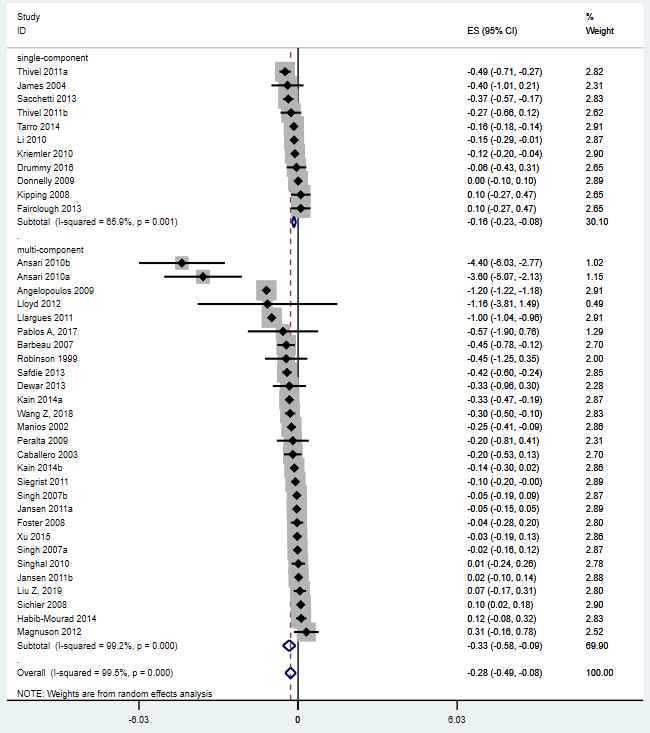


**Figure S2 Pooled intervention effect after excluding studies at high risk of bias (BMI)**


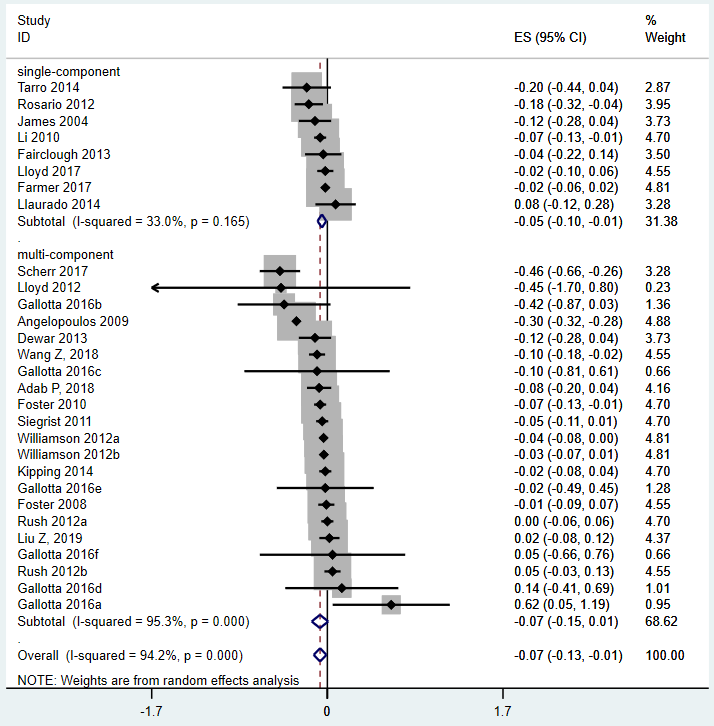


**Figure S3 Pooled intervention effect after excluding studies at high risk of bias (BMI Z-score)**

Fi

**Protocol of a systematic review and meta-analysis of school-based obesity prevention interventions**

- **Review question**
- How much are the overall effects of school-based obesity prevention interventions?
- What characteristics of intervention components are uniquely related to effectiveness in school-based obesity prevention interventions?
- **Eligibility criteria**
  - Study design

We will include individually or cluster-randomized controlled trials. We will not include non-randomized or uncontrolled trials.

- - Participants

We will include interventions implemented among students of elementary or secondary schools (aged 5~18 years).

- - Interventions

We will include interventions aiming for promoting healthy weight or prevention of overweight or obesity. We will not include interventions aiming for treatment of overweight or obesity, or specially designed for the treatment of obesity-related diseases (e.g., type 2 diabetes or hypertension). The duration of intervention should be at least 3 months.

- - Comparators

We will include comparison groups being active controls, usual practice controls or wait-list controls.

- - Outcomes

We will include studies assessing students’ body mass index (BMI) or BMI Z-score. We will exclude studies using only questionnaires to collect the adiposity outcomes.

- - Other criteria

We will include English full-text publications after January 1990. We will exclude studies not providing statistics necessary for meta-analyses.

- **Searches**
  - Sources: MEDLINE, CENTRAL and Embase databases, as well as the reference lists of all retrieved full text reviews;
  - Search strategy (shown in the first part of this Supplementary Material).
- **Data extraction**

Two persons will independently extract data based on a unified worksheet. The collected information will include the authors; publication date; country; funding source; study design; eligibility criteria; characteristics of the study population (including age, gender); characteristics of interventions (sessions, dosage, duration, mode of delivery); types and contents of comparators; attrition rate; process data; outcomes and results. For results, we will collect outcome measures used, time of assessment and statistics (numbers of participants, means, standard deviations or other summary statistics). We will resolve any disagreements by discussion or consulting a third personnel.

- **Risk of bias assessment**

We will assess risk of bias of individual studies following the Cochrane guidance, including selection bias (random sequence generation, concealment of the allocation sequence), performance bias (blinding of participants and personnel), detection bias (blinding of outcome assessment), attrition bias (incomplete outcome data) and reporting bias (selective outcome reporting, publication bias), as well as bias related to the cluster analyses (inappropriate statistical method).

We will include all studies in the initial analyses, and then exclude studies being assessed as at high risk of bias. Each domain will be rated as having a high, low or unclear risk of bias.

- **Assessment of heterogeneity**

We will use the I^2^ statistic to provide a measure of heterogeneity. The level of heterogeneity across studies will be rated as low (I^2^ = 25%), moderate (I^2^ = 50%) or high (I^2^ = 75%). We will include all studies in the initial analyses, and then exclude any individual studies for which the 95% CI of the intervention effect does not overlap with others.

- **Assessment of reporting biases**

If a minimum of 10 studies will be included, we will draw funnel plots to assess publication bias.

- **Data synthesis**

As the effect sizes of single- and multi-component interventions have been detected significant differences, we will report the synthesis results after classifying the included interventions into these two groups. As we expect a considerable amount of heterogeneity across included studies, we will use the random-effects model to pool the results by inverse variance methods. Our main analyses will be at the end of intervention. We will use Stata/SE 15.0 (StataCorp) for all analyses.

- **Subgroup analyses**

To identify the characteristics of interventions potentially contributing to the improved effects, we will first screen the studies by using “effectiveness ratio” (the ratio of intervention components used in effective trials relative to those used in both effective and non-effective trials) and select the potentially effective characteristics. Then we will use subgroup meta-analyses to compare the pooled results of those including these potentially effective characteristics vs. those not.

- **Sensitivity analyses**

If we identify moderate or high levels of heterogeneity in the meta-analyses, we will exclude any individual studies for which the 95% CI of the intervention effect does not overlap with others. We will also compare the pooled results obtained by all studies with those excluding individual studies at high risk of bias.

**References**

1. Drummy C, Murtagh EM, McKee DP, et al. The effect of a classroom activity break on physical activity levels and adiposity in primary school children. J Paediatr Child Health. 2016;52:745-9.

2. Kriemler S, Zahner L, Schindler C, et al. Effect of school based physical activity programme (KISS) on fitness and adiposity in primary schoolchildren: cluster randomised controlled trial. BMJ. 2010;340:c785.

3. Thivel D, Isacco L, Lazaar N, et al. Effect of a 6-month school-based physical activity program on body composition and physical fitness in lean and obese schoolchildren. Eur J Pediatr. 2011;170:1435-43.

4. Aguilar FS, Martínez-Vizcaíno V, López MS, et al. Impact of an after-school physical activity program on obesity in children. J Pediatr. 2010;157:36-42.

5. Donnelly JE, Greene JL, Gibson CA, et al. Physical Activity Across the Curriculum (PAAC): a randomized controlled trial to promote physical activity and diminish overweight and obesity in elementary school children. Prev Med. 2009;49:336-41.

6. Yan-Ping LI, Xiao-Qi HU, Schouten EG, et al. Report on childhood obesity in China (8): effects and sustainability of physical activity intervention on body composition of Chinese youth. Biomed Environ Sci. 2010;23:180-7.

7. Sacchetti R, Ceciliani A, Garulli A, et al. Effects of a 2‐year school‐based intervention of enhanced physical education in the primary school. J Sch Health. 2013;83:639-46.

8. Lloyd J, Creanor S, Logan S, et al. Effectiveness of the Healthy Lifestyles Programme (HeLP) to prevent obesity in UK primary-school children: a cluster randomised controlled trial. Lancet Child Adolesc Health. 2018. 2(1):35-45.

9. Llauradó E, Tarro L, Moriña D, et al. EdAl-2 (Educacio en Alimentacio) programme: reproducibility of a cluster randomised, interventional, primary-school-based study to induce healthier lifestyle activities in children. BMJ open. 2014;4:e005496.

10. Tarro L, Llauradó E, Albaladejo R, et al. A primary-school-based study to reduce the prevalence of childhood obesity–the EdAl (Educació en Alimentació) study: a randomized controlled trial. Trials. 2014;15:58.

11. Kipping R, Payne C, Lawlor DA. Randomised controlled trial adapting American school obesity prevention to England. Arch Dis Child. 2008;93:469-73.

12. James J, Thomas P, Cavan D, et al. Preventing childhood obesity by reducing consumption of carbonated drinks: cluster randomised controlled trial. BMJ. 2004;328:1237.

13. Fairclough SJ, Hackett AF, Davies IG, et al. Promoting healthy weight in primary school children through physical activity and nutrition education: a pragmatic evaluation of the CHANGE! randomised intervention study. BMC public health. 2013;13:626.

14. Rosário R, Oliveira B, Araújo A, et al. The impact of an intervention taught by trained teachers on childhood overweight. Int J Environ Res Public Health. 2012;9:1355-67.

15. Farmer VL, Williams SM, Mann JI, et al. The effect of increasing risk and challenge in the school playground on physical activity and weight in children: a cluster randomised controlled trial (PLAY). Int J Obes. 2017;41:793.

16. Hollis JL, Sutherland R, Campbell L, et al. Effects of a‘school-based’physical activity intervention on adiposity in adolescents from economically disadvantaged communities: secondary outcomes of the ‘Physical Activity 4 Everyone’ RCT. Int J Obes. 2016;40:1486.

17. El Ansari W, El Ashker S, Moseley L. Associations between physical activity and health parameters in adolescent pupils in Egypt. Int J Environ Res Public Health. 2010;7:1649-69.

18. Robinson TN. Reducing children's television viewing to prevent obesity: a randomized controlled trial. JAMA. 1999;282:1561-7.

19. Sichieri R, Trotte AP, de Souza RA, et al. School randomised trial on prevention of excessive weight gain by discouraging students from drinking sodas. Public Health Nutr. 2009;12:197-202.

20. Williamson DA, Champagne CM, Harsha DW, et al. Effect of an environmental school‐based obesity prevention program on changes in body fat and body weight: a randomized trial. Obesity. 2012;20:1653-61.

21. Llargues E, Franco R, Recasens A, et al. Assessment of a school-based intervention in eating habits and physical activity in school children: the AVall study. J Epidemiol Community Health. 2011;65:896-901.

22. Jansen W, Borsboom G, Meima A, et al. Effectiveness of a primary school-based intervention to reduce overweight. Int J Pediatr Obes. 2011;6:e70-77.

23. Manios Y, Moschandreas J, Hatzis C, et al. Health and nutrition education in primary schools of Crete: changes in chronic disease risk factors following a 6-year intervention programme. Br J Nutr. 2002;88:315-24.

24. Peralta LR, Jones RA, Okely AD. Promoting healthy lifestyles among adolescent boys: the Fitness Improvement and Lifestyle Awareness Program RCT. Prev Med. 2009;48:537-42.

25. Vandongen R, Jenner DA, Thompson C, et al. A controlled evaluation of a fitness and nutrition intervention program on cardiovascular health in 10-year-old to 12-year-old children. Prev Med. 1995;24:9-22.

26. Siegrist M, Hanssen H, Lammel C, et al. Effects of the school-based intervention programme JuvenTUM 3 on physical fitness, physical activity, and the prevalence of overweight. Eur J Prev Cardiolog. 2012;19:S2.

27. Jiang J, Xia X, Greiner T, et al. The effects of a 3‐year obesity intervention in schoolchildren in Beijing. Child Care Health Dev. 2007;33:641-6.

28. Magnusson KT, Hrafnkelsson H, Sigurgeirsson I, et al. Limited effects of a 2-year school-based physical activity intervention on body composition and cardiorespiratory fitness in 7-year-old children. Health Educ Res. 2012;27:484-94.

29. Dewar DL, Morgan PJ, Plotnikoff RC, et al. The nutrition and enjoyable activity for teen girls study: a cluster randomized controlled trial. Am J Prev Med. 2013;45:313-7.

30. HEALTHY Study Group. A school-based intervention for diabetes risk reduction. N Engl J Med. 2010;363:443-53.

31. Angelopoulos PD, Milionis HJ, Grammatikaki E, et al. Changes in BMI and blood pressure after a school based intervention: the CHILDREN study. Eur J Public Health. 2009;19:319-25.

32. Barbeau P, Johnson MH, Howe CA, et al. Ten months of exercise improves general and visceral adiposity, bone, and fitness in black girls. Obesity. 2007;15:2077-85.

33. Lloyd JJ, Wyatt KM, Creanor S. Behavioural and weight status outcomes from an exploratory trial of the Healthy Lifestyles Programme (HeLP): a novel school-based obesity prevention programme. BMJ open. 2012;2:e000390.

34. Xu F, Ware RS, Leslie E, Tse LA, Wang Z, Li J, Wang Y. Effectiveness of a randomized controlled lifestyle intervention to prevent obesity among Chinese primary school students: CLICK-obesity study. PLoS One. 2015 Oct 28;10(10):e0141421.

35. Kipping R, Howe L, Jago R, et al. Effect of intervention aimed at increasing physical activity, reducing sedentary behaviour, and increasing fruit and vegetable consumption in children: active for life year 5 (AFLY5) school based cluster randomised controlled trial. BMJ. 2014;348:3256.

36. Scherr RE, Linnell JD, Dharmar M, et al. A multicomponent, school-based intervention, the Shaping Healthy Choices Program, improves nutrition-related outcomes. J Nutr Educ Behav. 2017;49:368-79.

37. Habib-Mourad, Carla. An intervention to promote healthy eating and physical activity in Lebanese school children : Health-E-PALS : a pilot cluster randomised controlled trial. Durham University. 2013

38. Foster GD, Sherman S, Borradaile KE, et al. A policy-based school intervention to prevent overweight and obesity. Pediatrics. 2008;121:e794-802.

39. Rush E, Reed P, McLennan S, et al. A school-based obesity control programme: Project Energize. Two-year outcomes. Br J Nutr. 2012;107:581-7.

40. Safdie M, Jennings-Aburto N, Lévesque L, et al. Impact of a school-based intervention program on obesity risk factors in Mexican children. Salud Publica Mex. 2013;55:374-87.

41. Gallotta MC, Iazzoni S, Emerenziani GP, et al. Effects of combined physical education and nutritional programs on schoolchildren’s healthy habits. PeerJ. 2016;4:e1880.

42. Kain J, Concha F, Moreno L, et al. School-based obesity prevention intervention in Chilean children: effective in controlling, but not reducing obesity. J Obes. 2014;2014.

43. Caballero B, Clay T, Davis SM, et al. Pathways: a school-based, randomized controlled trial for the prevention of obesity in American Indian schoolchildren. Am J Clin Nutr. 2003;78:1030-8.

44. Singhal N, Misra A, Shah P, et al. Effects of controlled school-based multi-component model of nutrition and lifestyle interventions on behavior modification, anthropometry and metabolic risk profile of urban Asian Indian adolescents in North India. Eur J Clin Nutr. 2010;64:364.

45. Singh AS, Paw MJ, Brug J, et al. Short-term effects of school-based weight gain prevention among adolescents. Arch Pediatr Adolesc Med. 2007;161:565-71.

46. Grydeland M, Bjelland M, Anderssen SA, et al. Effects of a 20-month cluster randomised controlled school-based intervention trial on BMI of school-aged boys and girls: the HEIA study. Br J Sports Med. 2014;48:768-73.

47. Magnusson KT, Sigurgeirsson I, Sveinsson T, et al. Assessment of a two-year school-based physical activity intervention among 7-9-year-old children. Int J Behav Nutr Phys Act. 2011;8:138.

48. Lubans DR, Morgen PJ, Okely AD, et al. Preventing Obesity Among Adolescent Girls: One-Year Outcomes of the Nutrition and Enjoyable Activity for Teen Girls (NEAT Girls) Cluster Randomized Controlled Trial. Arch Pediatr Adolesc Med. 2012; 166:821-7.

49. Campbell R, Rawlins E, Wells S, et al. Intervention fidelity in a school-based diet and physical activity intervention in the UK: Active for Life Year 5. Int J Behav Nutr Phys Act. 2015; 12:141.

50. Grydeland M, Bergh IH, Bjelland M, et al. Intervention effects on physical activity: the HEIA study - a cluster randomized controlled trial. Int J Behav Nutr Phys Act. 2013;10:17.
